# Supplementary material for: PABPN1 loss-of-function causes APA-shift in oculopharyngeal muscular dystrophy
Source: HGG Adv. 2024 Jan 11;5(2):100269. doi: 10.1016/j.xhgg.2024.100269 (PMC10840355; doi:10.1016/j.xhgg.2024.100269)
Supplement: Document S1. Tables S1, S2 and S5 and Figures S1–S4 [file mmc1.pdf]

HGGA, Volume *5*

Supplemental information

PABPN1 loss-of-function causes APA-shift in oculopharyngeal muscular dystrophy

Milad Shademan, Hailiang Mei, Baziel van Engelen, Yavuz Ariyurek, Susan Kloet, and Vered Raz

Supplementary data

A. Supplementary Tables

Table S1: Gender and age information of OPMD and control subjects whose RNA from vastus lateralis muscle was used in this study.

OPMD			Control		
Sample nr	**Age range**	**Gender**	Sample nr	**Age range**	**Gender**
1	60-69	f	3	20-29	m
2	40-49	m	4	60-69	m
3	50-59	m	5	60-69	f
4	80-89	f	6	80-89	f
5	40-49	f	7	30-39	m
6	50-59	f	8	40-49	f
7	40-49	f	11	80-49	f
8	70-79	m	12	50-59	m
9	60-69	f			
10	40-49	f			

Table S2: Data sets description

A. Experiment	Sample nr.	Library prep	Transcript exclusion criteria	Transcript nr. (3'UTR)	Included transcript nr.	Transcript with APA shift
**Mouse (FVB and A17.1)**	5 + 5	7C	∑ reads proximal =<10 and	52218	24106	3728
	5 + 5	1C			14111	1507
**Mouse cell line (A10 and A17)**	3 + 3	7C			23869	753
	3 + 3	1C			8278	258
**Human (control and OPMD)**	8 + 10	1C	Reads at proximal region in = > 30% of samples	85619	3113	215

**B.**	**Category**	**Total input reads**	**Unmapped reads**		**Mapped reads**		**Reads after UMI based dedup (transcriptome based approach)**	
**7C**	FVB_Q2	12404400	337395	2.72%	12067005	97.28%	6939372	57.51%
	FVB_Q3	13815810	342575	2.48%	13473235	97.52%	7080216	52.55%
	FVB_Q4	12525017	301601	2.41%	12223416	97.59%	8004434	65.48%
	FVB_Q5	13073935	320279	2.45%	12753656	97.55%	7985539	62.61%
	FVB_Q6	11424354	297712	2.61%	11126642	97.39%	7445899	66.92%
	A17.1_Q2	11229458	315988	2.81%	10913470	97.19%	6485672	59.43%
	A17.1_Q3	10951353	310997	2.84%	10640356	97.16%	5586852	52.51%
	A17.1_Q4	11602273	334719	2.88%	11267554	97.12%	6286475	55.79%
	A17.1_Q5	10327784	294344	2.85%	10033440	97.15%	5854875	58.35%
	A17.1_Q6	10357987	319769	3.09%	10038218	96.91%	6209008	61.85%
	A10_1	14786997	514065	3.48%	14272932	96.52%	10443091	73.17%

	A10_2	9284162	321864	3.47%	8962298	96.53%	6911140	77.11%
	A10_3	9044039	292607	3.24%	8751432	96.76%	6568761	75.06%
	A17_1	10643513	388054	3.65%	10255459	96.35%	7073487	68.97%
	A17_2	11148411	370948	3.33%	10777463	96.67%	8018058	74.40%
	A17_3	11290889	341323	3.02%	10949566	96.98%	7653099	69.89%
**1C**	FVB_Q2	1550669	475119	30.64%	1075550	69.36%	871531	81.03%
	FVB_Q3	1816421	546595	30.09%	1269826	69.91%	960845	75.67%
	FVB_Q4	1635290	494250	30.22%	1141040	69.78%	982341	86.09%
	FVB_Q5	2274844	696713	30.63%	1578131	69.37%	1316496	83.42%
	FVB_Q6	2268734	698919	30.81%	1569815	69.19%	1344212	85.63%
	A17.1_Q2	1418780	443039	31.23%	975741	68.77%	791623	81.13%
	A17.1_Q3	932978	289091	30.99%	643887	69.01%	498184	77.37%
	A17.1_Q4	1606702	483546	30.10%	1123156	69.90%	878087	78.18%
	A17.1_Q5	1716088	525516	30.62%	1190572	69.38%	941188	79.05%
	A17.1_Q6	1638100	507705	30.99%	1130395	69.01%	930914	82.35%
	A10_1	556663	146303	26.28%	410360	73.72%	364145	88.74%
	A10_2	484524	131897	27.22%	352627	72.78%	315090	89.36%
	A10_3	355590	104663	29.43%	250927	70.57%	223657	89.13%
	A17_1	153917	44902	29.17%	109015	70.83%	97100	89.07%
	A17_2	280383	78810	28.11%	201573	71.89%	179363	88.98%
	A17_3	530802	147575	27.80%	383227	72.20%	338299	88.28%
**Control subjects**	c11	2117733	1824795	86.17%	292938	13.83%	92274	31.50%
	c12	4182735	3391372	81.08%	791363	18.92%	236127	29.84%
	c3	1840311	1558919	84.71%	281392	15.29%	81990	29.14%
	c4	2267725	1903233	83.93%	364492	16.07%	84056	23.06%
	c5	1686682	1388817	82.34%	297865	17.66%	84010	28.20%
	c6	2198539	1861224	84.66%	337315	15.34%	126287	37.44%
	c7	1467447	1329313	90.59%	138134	9.41%	70051	50.71%
	c8	3574491	3171647	88.73%	402844	11.27%	179056	44.45%
**OPMD subjects**	p1	4236462	3618811	85.42%	617651	14.58%	179128	29.00%
	p2	3269827	2851317	87.20%	418510	12.80%	140022	33.46%
	p3	2399867	2073306	86.39%	326561	13.61%	101192	30.99%
	p4	2868232	2414007	84.16%	454225	15.84%	124061	27.31%
	p5	3371018	2689161	79.77%	681857	20.23%	176315	25.86%
	p6	1674483	1437591	85.85%	236892	14.15%	89196	37.65%
	p7	3700454	3243308	87.65%	457146	12.35%	158209	34.61%
	p8	6500730	5446068	83.78%	1054662	16.22%	397067	37.65%
	p9	2386840	1875333	78.57%	511507	21.43%	159734	31.23%
	p10	2849503	2313485	81.19%	536018	18.81%	137917	25.73%

C.

SCIFI_LIG384	GAGTTCAGACGTGTGCTCTTCCGATCT-NNNNNNNN-AAGTGATTAGCAA-TTTTTTTTTTTTTTTTTTTTTTTTTTTTTTVN
Well barcode	CAAGCAGAAGACGGCATACGAGAT[i7_barcode]GTGACTGGAGTTCAGACGTGTGCTCTTCCGATCT)

**D.**	Forward 5' – 3'	Reverse 5' – 3'

Exon 4-5<br>ENSG00000100836	ATGTTGGCAATGTGGACTATG	ACACGGTTGACTGAACCACA
Iso-201<br>ENST00000216727	GTTTTAACAGCAGGCCCCG	TCTTTTTTTCCTCTCTCTCCTCCTAATAC
Iso-202<br>ENST00000397276	CAACAGCCTTGTGGGAGGAT	CAAAAACCTGGGCACCACAC
Iso-207<br>ENST00000556821	CCCGACTGGCTTGATTCGG	CATGCTCGGCCATTTCCCT
HPRT1	TGGTCAGGCAGTATAATCCAAAGA	TCAAATCCAACAAAGTCTGGCTTA

A. Summary of data sets that were used in this study, transcripts' number that was used for APA-shift calculation and the number of significant APA-shift transcripts in each experiment.

B. RNAseq quality control in 7C and 1C protocols in OPMD mouse and cell line models, and in human muscles (controls and OPMD).

C. Primers used for library preparation.

D. Primers used for RT-qPCR

Table S3: Transcripts with APA-shift in mouse, cell line and human samples.

Table S4: List of muscle transcripts that were included in this study.

Table S5: expression levels of PABPN1 transcript isoforms in mouse and cell models.

Mouse Tibialis anterior

Transcript	Isoform	FVB_1	FVB_2	FVB_3	FVB_4	FVB_5	A17_1	A17_2	A17_3	A17_4	A17_5
ENSMUST00000022808	Iso-201	31.03	18.70	28.09	32.55	25.88	9.99	0.00	12.37	14.89	14.41
ENSMUST00000172557	Iso-208	34.75	23.04	29.19	34.23	23.16	13.58	0.00	10.49	18.69	11.31
ENSMUST00000116476	Iso-202	9.90	18.48	9.22	7.19	11.92	28.76	23.21	18.27	13.30	19.96
ENSMUST00000150975	Iso-207	4.95	11.95	8.06	4.49	10.21	20.37	19.34	14.21	10.45	13.65
ENSMUST00000172695	Iso-209	19.88	28.27	33.48	24.38	21.28	18.97	14.89	12.97	16.03	15.59

Muscle cell culture

Transcript	Isoform	WTA_1	WTA_2	WTA_3	D7E_1	D7E_2	D7E_3
ENSMUST00000022808	Iso-201	47.84	27.89	39.15	95.27	59.93	32.02
ENSMUST00000172557	Iso-208	60.77	32.16	30.19	113.91	65.50	42.11
ENSMUST00000116476	Iso-202	34.48	14.45	48.62	27.16	33.41	47.06
ENSMUST00000150975	Iso-207	25.08	14.45	24.31	18.10	27.84	43.70
ENSMUST00000172695	Iso-209	37.61	61.40	48.62	27.16	83.52	43.70

B. Supplementary figures

Figure S1: A flow-chat summary of the bioinformatic steps from RNA-seq to APA-shift calculation

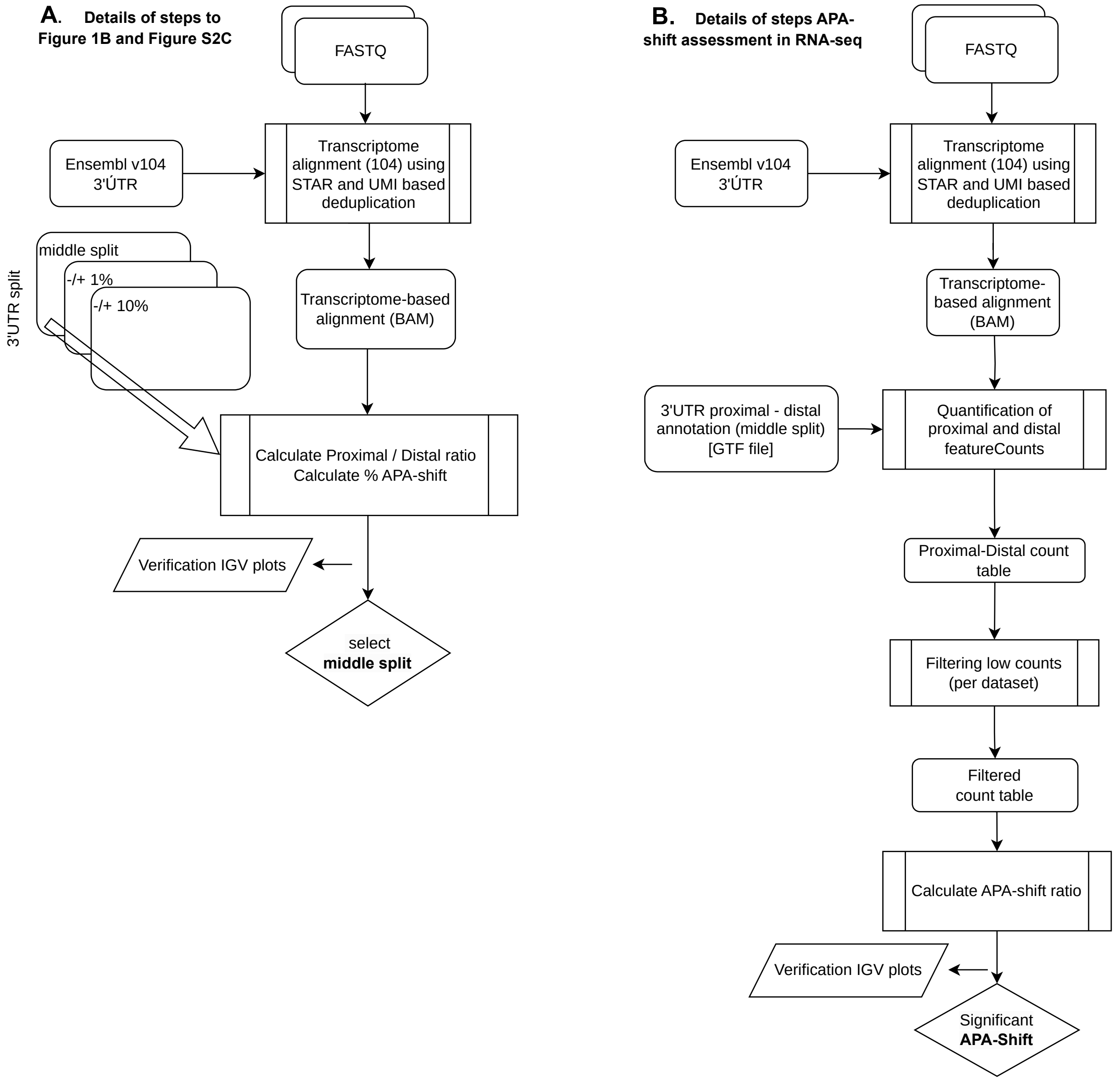

A. A flowchart of the steps to select the middle split in the 3'-UTR for APA-shift calculation. Results are in Fig. 1B and Figure S2C.

B. A flowchart of the steps to calculate APA shift from RNA-seq. The codes are in GIT.

Figure S2. 1C vs. 7C library prep results in mouse and cell OPMD models

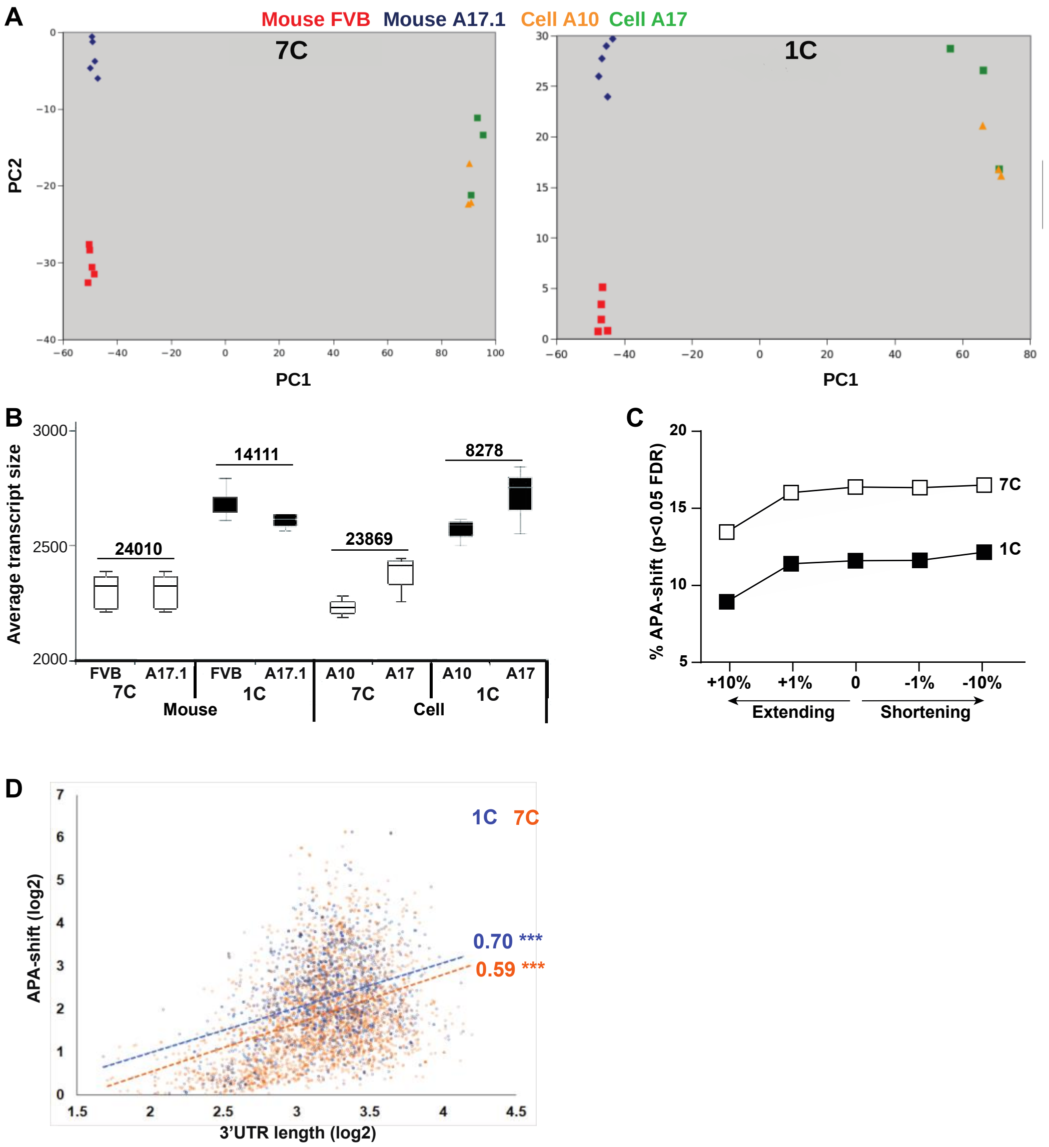

A. PCA plots of 7C or 1C RNAseq in mouse (FVB; A17.1) or mouse cells (A10; A17).

B. Boxplot of 7C and 1C average insert size in mouse and cell models. The number of transcripts passing exclusion criteria is depicted.

C. The percentage of transcripts with APA-shift (p<0.05, FDR) in mouse samples using different separation proximal and distal regions at the 3'UTR. 7C is denoted with open squares and 1C with close squares.

D. Dot plot of 3'UTR length vs APA-shift (log2) of transcripts with APA-shift<0.05, FDR, in 7C and 1C datasets. The linear regression line is depicted with a dashed line, and the slope and significance (*** p<0.0001) are shown next to the slope.

Figure S3. 1C vs. 7C APA-seq in an OPMD muscle cell model

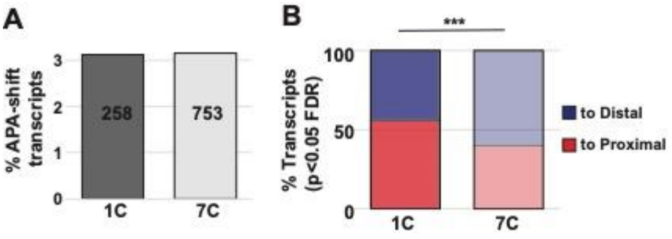

A. Bars show the percentage of transcripts with APA-shift from total transcripts in 1C or 7C datasets.

B. Bars show percentage of transcripts proximal (red) or distal (blow) APA-shift from the significant ($p<0.05$, FDR) APA-shift transcripts in 1C or 7C datasets. A statistical difference between 1C and 7C shift direction was assessed with the chi-square test; *** $p<0.0001$.

Figure S4: IGV and PAS-seq comparison in six transcripts

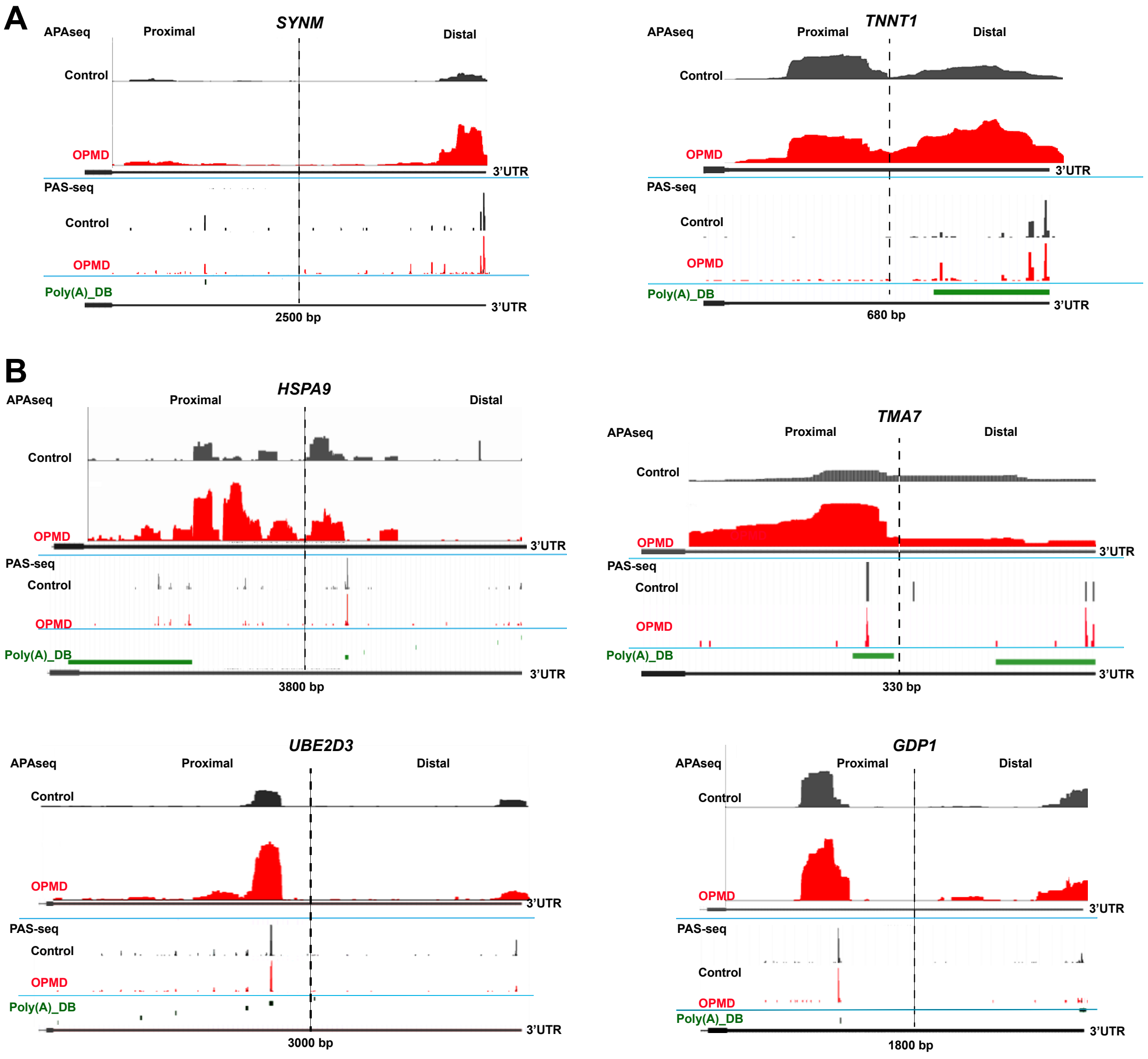

Transcripts examples are for shift to distal (A) or shift to proximal (B). Fer transcript, IGV in control (black) and OPMD (red) are on top followed by PAS-seq and PAS from poly(A)_DB (in green). The ensemble annotation 3'UTR is at the bottom, the length of the 3'UTR is depicted and a dashed line separates the proximal from distal regions.

Figure S5. PCA plots of APA-shift values in 1C samples

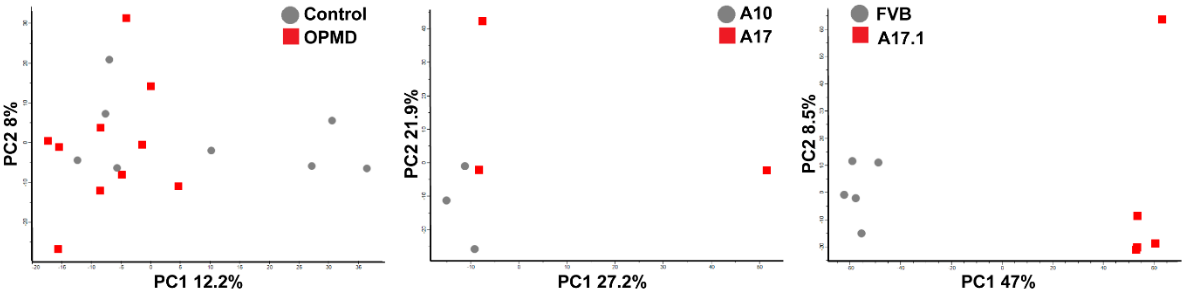

From left to right: human (controls and OPMD), muscle cell models (A10 and A17) , and mouse model (FVB and A17.1). The percentage of variance for the first two principal components (PC) is depicted.
