# Supplementary material for: PABPN1 loss-of-function causes APA-shift in oculopharyngeal muscular dystrophy
Source: HGG Adv. 2024 Jan 11;5(2):100269. doi: 10.1016/j.xhgg.2024.100269 (PMC10840355; doi:10.1016/j.xhgg.2024.100269)
Supplement: Document S2. Article plus supplemental information [file mmc4.pdf]

HGG Advances

ARTICLE

PABPN1 loss-of-function causes APA-shift in oculopharyngeal muscular dystrophy

Milad Shademan,[1,4] Hailiang Mei,[2,4] Baziel van Engelen,[3] Yavuz Ariyurek,[1] Susan Kloet,[1] and Vered Raz[1,5,*]

Summary

Alternative polyadenylation (APA) at the 3′ UTR of transcripts contributes to the cell transcriptome. APA is suppressed by the nuclear RNA-binding protein PABPN1. Aging-associated reduced PABPN1 levels in skeletal muscles lead to muscle wasting. Muscle weakness in oculopharyngeal muscular dystrophy (OPMD) is caused by short alanine expansion in PABPN1 exon1. The expanded PABPN1 forms nuclear aggregates, an OPMD hallmark. Whether the expanded PABPN1 affects APA and how it contributes to muscle pathology is unresolved. To investigate these questions, we developed a procedure including RNA library preparation and a simple pipeline calculating the APA-shift ratio as a readout for PABPN1 activity. Comparing APA-shift results to previously published PAS utilization and APA-shift results, we validated this procedure. The procedure was then applied on the OPMD cell model and on RNA from OPMD muscles. APA-shift was genome-wide in the mouse OPMD model, primarily affecting muscle transcripts. In OPMD individuals, APA-shift was enriched with muscle transcripts. In an OPMD cell model APA-shift was not significant. APA-shift correlated with reduced expression levels of a subset of PABPN1 isoforms, whereas the expression of the expanded PABPN1 did not correlate with APA-shift. PABPN1 activity is not affected by the expression of expanded PABPN1, but rather by reduced PABPN1 expression levels. In muscles, PABPN1 activity initially affects muscle transcripts. We suggest that muscle weakness in OPMD is caused by *PABPN1* loss-of-function leading to APA-shift that primarily affects in muscle transcripts.

Introduction

The landscape of mRNAs in eukaryote cells is highly affected by the 3′ UTR of transcripts. The length of the 3′ UTR affects multiple layers of mRNA processing, including nuclear export, translation efficiency, and stability or decay.[1,2] The 3′ UTR length is determined by cleavage of the polyadenylation site (PAS); a long 3′ UTR is generated from a distal PAS, whereas utilization of proximal PAS generates the short isoform. Approximately 70% of genes have two or more PASs at the 3′ UTR.[3] PASs can be discriminated between canonical and alternative PASs (APAs), and from the latter, alternative transcripts are formed.[3,4] In general, longer 3′ UTR transcripts have higher translation efficiency,[5] whereas short 3′ UTR have reduced abundance in the heavy polysomes and reduced translation efficiency.[6] APA generates alternative transcripts that augment the cell transcriptome and protein landscape.

APA is regulated by RNA processing and RNA-binding proteins.[7,8] Some are ubiquitously expressed and others have a cell- or tissue-specific expression.[9,10] Consequently, alternative transcripts generated from APA can show a cell-type-specific pattern and can affect biological processes in health and disease.[7,11]

Among the RNA-binding proteins regulating APA site utilization, the polyadenosine (poly(A)) RNA-binding protein nuclear1 (PABPN1, OMIM: 3602279) suppresses the APA. PABPN1 depletion causes genome-wide APA site utilization and shorter transcripts.[3,12] These shorter transcripts have a lower abundance in polyribosomes, affecting translation efficiency.[6]

PABPN1 is a vital protein and is ubiquitously expressed in all cells. PABPN1 levels are reduced during normal aging in skeletal muscles.[13] Reduced PABPN1 levels were also reported in oculopharyngeal muscular dystrophy (OPMD), an adult-onset autosomal dominant myopathy. OPMD is caused by a trinucleotide alanine expansion mutation in the first exon of the gene encoding PABPN1.[14] The expanded PABPN1 forms nuclear aggregates and entraps other nuclear proteins and mRNAs, a histopathological mark of OPMD.[15] A mouse model overexpressing the expanded PABPN1 in the skeletal muscles exhibits muscle wasting,[16] concomitant with genome-wide APA alterations.[3,12] This mouse model was instrumental to demonstrate that PABPN1 aggregation leads to muscle pathology concurrently with APA. So far, genome-wide APA has not been reported in OPMD individuals, and it is unclear to what extent PABPN1 activity contributes to OPMD pathology.

To address this question, we developed a wet lab and computational procedure reporting changes in 3′ UTR length of mRNA isoforms called APA-shift. We show that this method is highly robust and specifically beneficial to detect APA-shift in muscle transcripts. Applying the

[1]Department of Human Genetics, Leiden University Medical Centre, Leiden, the Netherlands; [2]Department of Biomedical Data Sciences, Leiden University Medical Centre, Leiden, the Netherlands; [3]Department of Neurology, Radboud University Medical Centre, Nijmegen, the Netherlands
[4]These authors contributed equally
[5]Lead contact
*Correspondence: v.raz@lumc.nl
https://doi.org/10.1016/j.xhgg.2024.100269.
© 2024 The Author(s). This is an open access article under the CC BY license (http://creativecommons.org/licenses/by/4.0/).

method to OPMD models and to RNA from OPMD individuals we demonstrate that APA-shift correlates with reduced levels of PABPN1 isoform lacking the polyalanine expansion rather than by the expression of mutant PABPN1.

Materials and methods

Mouse

Tibialis anterior muscles from A17.1 and FVB males 18 months old were collected, stored, and bulk RNA was extracted as previously reported.[16]

Mouse muscle cells

Stable cells expressing Ala10 or Ala17 PABPN1 under muscle actin promoter were reported.[17] Bulk RNA was extracted from fused cultures as detailed by Raz et al.[17]

Human

Vastus lateralis muscle was collected from control and OPMD subjects, genetically confirmed, and previously detailed.[18] OPMD individuals were recruited via the Dutch neuromuscular database (Computer Registry of All Myopathies and Polyneuropathies). Twelve controls and 10 OPMD subjects were included in this study. Four controls were excluded due to poor sequencing quality. Age range and gender of OPMD and controls subjects are listed in Table S1.

All of the RNAs were stored at −80°C and RNA integrity was determined before cDNA library preparation. The samples and models that were used for RNA sequencing (RNA-seq) and downstream analysis are listed in Table S2A.

Library preparation and RNA-seq

The generation of cDNA for the RNA-seq data was carried out with the mcSCRB-seq protocol,[19] with the following modifications to the workflow: instead of single cells, we used 1–100 ng isolated total RNA, 7.5% PEG8000 was replaced with 0.5% polyvinylpyrrolidone, and the oligo-dT primer was replaced with the SCIFI_LIG384_001 primer (Table S2C, also described in Datlinger et al.[20]).

All samples were barcoded during reverse transcription using a well barcode (Table S2C). After reverse transcription, the full-length cDNA was either unamplified (1C) or underwent 7 cycles of amplification (7C) using 2× Kapa HiFi HotStart mix (Roche). cDNA was then pooled, and sequencing libraries were generated using the Kapa Hyper Plus kit (Roche) using a standard protocol. Because the 1C libraries were not preamplified, we added more cycles, namely 14 cycles, and the p7 primer was replaced with i7 Illumina barcode per pool. After an additional double solid-phase reversible immobilization size selection, the Illumina library was quantified using Qubit and checked on an Agilent Lab-on-a-Chip for the size distribution. Illumina paired-end sequencing of the library was done on a NovaSeq 6000 using paired-end 150-bp sequencing and v1.5 chemistry. After sequencing, read 1 will contain the cDNA information and read 2 will contain only the unique molecular identifier (UMI).

For the pipeline to run effortlessly, the UMI in the read 2 FASTQ file was added to the beginning of the same read identifier in the read 1 FASTQ file. The modified read 1 FASTQ file starts with the UMI and is followed by the cDNA sequence, which was used in the RNA-seq pipeline. For the demultiplexing the pooled samples we used the well barcode from the oligodT primer using Ultraplex (https://github.com/ulelab/ultraplex).[21] A summary of quality control analysis of all of the samples is found in Table S2B.

RNA-seq analysis and APA-shift calculation

To process the RNA-seq data to establish the APA-shift calculation in both our human and mouse studies, we established a data preprocessing pipeline (Figure S1). All of the mouse and human reads in FASTQ format are first filtered using Cutadapt (version 2.10) to remove all of the remaining adapter sequences. Then, the reads were aligned to the Ensembl transcriptome version 104 using STAR (version 2.7.5a) including UMI-based deduplication using UMI-Tools (version 1.1.1) to generate transcriptome-based alignment in binary alignment map (BAM) format so that we could visualize using the Integrative Genomics Viewer (IGV). By inspecting these alignment files in 3′ UTR using IGV, we could observe clear enriched signals indicating both proximal and distal APA sites (Figure 1A). Confirmed by the visual inspection in IGV, we used the same Ensembl gene annotation version 104 to create a customized 3′ UTR annotation gene transfer format (GTF) file for all Ensembl transcripts with annotated 3′ UTR. In this GTF file, 3′ UTRs are split in the middle to create a proximal and a distal region. With this APA proximal and distal annotation file and the human and mouse transcriptome-based alignment file as input, we quantified the APA enrichment signal at both proximal and distal regions using featureCounts (version 2.0.1) with option "-M" to include multiple mapped reads.

Transcripts were excluded according to the exclusion criteria in Table S2. Raw counts were used to calculate the proximal to distal ratio (P/D) in R, using the following equation: $\log(1+P) - \log(1+D)$. APA-shift, in a log scale, was calculated between OPMD and control as P/D [OPMD] − P/D[control]. A ratio larger than 0 indicates a shift to proximal and smaller than 0 a shift to distal. Statistical significance for APA-shift was considered with a $p < 0.05$ corrected for false discovery rate (FDR). Codes for the bioinformatics pipeline are found in https://github.com/lumc-sasc/pub-PABPN1_APA-shift_Raz.

For human data, we incorporated age and gender as covariates in a model matrix. The P/D ratios were corrected on data corrected for age and gender using a linear model in 'limma,' and APA-shift was calculated on the corrected data. A list of transcripts with APA-shift in mouse, cell, and human samples is found in Table S3). Visualization of results was carried out with ggvolcanoR.[22]

To assess whether splitting the 3′ UTR in the middle is a robust choice, we examined APA-shift results by extending or shortening the annotated 3′ UTR. Four different shortening or extension factors were examined. A shortening refers to moving the starting position of 3′ UTR toward the 3′ end, whereas extension refers to moving the starting position of 3′ UTR toward the 5′ end. The 3′ end position of a transcript was unchanged. The resulted shortened or extended 3′ UTR were split half and half, defining the proximal or distal region. Per direction, 10% and 1% moving factors were analyzed. Counts normalized to the mean were used to calculate expression levels.

qRT-PCR

qRT-PCR was conducted on RNA extracted from the control and OPMD muscles. For cDNA synthesis, 500 ng RNA was reverse transcribed using the QuantiTect Reverse Transcription Kit (QIAGEN) and random primers, following the manufacturer's instructions. Subsequently, qPCR amplification was performed with the

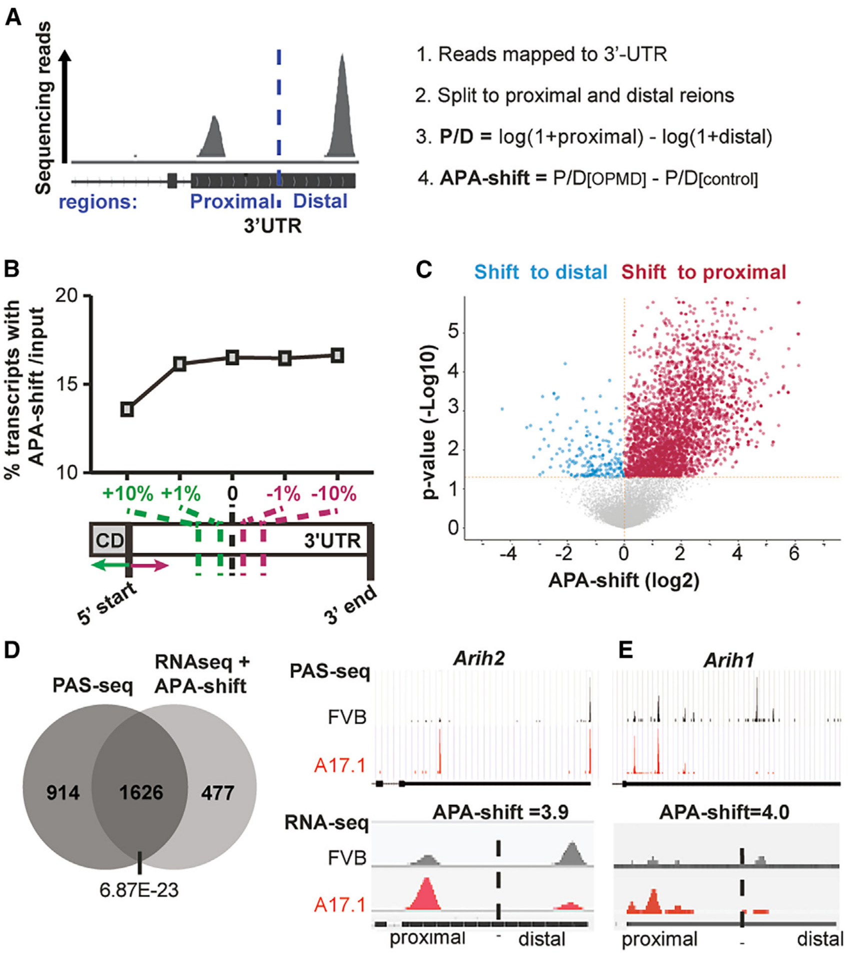

Figure 1. APA-shift calculation
(A) (Left) IGV from a 3′ UTR transcript example for the RNA-seq. The split point between proximal and distal regions is denoted with a dashed line. (Right) A summary of major steps in the APA-shift pipeline calculation.
(B) Shortening and extending split point 3′ UTR regions effect on APA-shift. A schematic presentation of 3′ UTR splitting into proximal and distal regions: in the middle (depicted by dashed black line), or extension toward 5′ sequences (in green) or shortening toward 3′ sequences (in red) by 1% and 10% relative to the middle split, are depicted under the dot plot. Dot plot shows the percentage of transcripts with APA-shift.
(C) Volcano plot of APA-shift in A17.1 versus FVB mouse using a generic protocol. Mean log fold change and p values are from n = 5 per genotype. Transcripts with a switch to proximal are highlighted in red and a switch to distal in blue.
(D) IGV plots of the sum of reads at the 3′ UTR for 2 example significant genes, *Arih2* and *Arih1*. 3′ UTR from PAS-seq (de Klerk et al.[3]) and APA-seq in FVB and A17.1. The 3′ UTR is depicted by the black horizontal bar, and the vertical dashed line indicates the 0 position. The average APA-shift fold change for *Arih1* ENSMUST00000171975 and *Arih2* ENSMUST00000013338 are depicted.
(E) Venn diagram shows the overlap of transcripts with significant APA-shift between PAS- and APA-seq. p value for overlap significance was determined with a chi-square test.

QuantiNova SYBR Green kit (QIAGEN) using 5 ng RNA, with technical duplicates, using a standard amplification protocol at a melting temperature of 60°C. Samples with Ct values above 35 were excluded from the analysis to eliminate potential noise. The average Ct values from the technical duplicates was used for ddCt calculation. PABPN1 Exon-4-5 (ENSG00000100836) was normalized to hypoxanthine ribosyltransferase, and isoforms 201 (ENST00000216727), 202 (ENST00000397276), and 207 (ENST00000556821) to exon 3–4. Isoforms 001 and 004 were targeted with specific primer sets, designed to cover different exons, thereby amplifying only mRNA molecules. Primer sets were designed with the NCBI Primer design tool (https://www.ncbi.nlm.nih.gov/tools/primer-blast/), and the primers sequence is in Table S2D.

Other resources

PAS-seq from A17.1 (5 + 5) and OPMD (4 OPMD individuals and 4 control subjects) muscles was generated as detailed by deKlerk et al.[3] BAM files of PAS-seq were used to visualize the genomic localization of reads peak. Transcripts with APA site utilization in A17.1 are from Table S2.[3]

Statistical and enrichment analyses

Statistical analyses were conducted in R 4.2.3 and GraphPad Prism 9.3.1. The statistical significance of APA-shift was assessed by applying the Benjamini-Hochberg method to adjust the p values for controlling the FDR.

The list of transcripts encoding for muscle proteins (called here muscle transcripts; Table S4) was downloaded from AmiGO2 Gene Ontology (GO) using the GO database (AmiGO 2 - GO Wiki, www.geneontology.org). Gene set enrichment analysis (GSEA)[23] version 4.3.2 was used for gene set enrichment analysis and DAVID[24] version 2023q1 for GO analysis considering the Bonferroni-corrected p value in human and A17.1 mice.

The visualization of reads at the 3′ UTR was carried out with the IGV version 2.16.0.

Results

APA-shift calculation captures real-time APA dynamics in muscle genes

Aiming to detect APA-shift in OPMD conditions, we developed a computational pipeline that quantifies global changes in PAS utilization at the 3′ UTR of transcripts from a standard poly(A) library preparation of the 3′ UTR. We generated libraries from the A17.1 mouse model, which overexpresses the expanded PABPN1 in skeletal muscles, and APA was previously demonstrated by others and us.[3,12] RNA-seq from these libraries was used to develop and optimize our pipeline. Since long transcripts

are generated from the distal PAS and short transcripts from a proximal PAS at the 3′ UTR, we defined proximal and distal regions based on a split in the middle of the 3′ UTR and calculated the ratio between the sum of reads from proximal and distal regions (Figure 1A). The P/D ratio (Figure 1A) and the ratio between OPMD conditions and control resulted in a calculated value we called APA-shift. We considered significant APA-shift with $p < 0.05$, FDR. Since multiple transcripts could belong to proximal and/or distal regions, and a split in the middle is an arbitrary choice, we assessed whether moving the 3′ UTR start point toward 5′ of the transcript (called extending) or toward 3′ of the transcript (called shortening) will affect global APA-shift results (Figure 1B). For the shortening by 1% or 10%, and the extension by 1%, after APA-shift recalculation in all 3′ UTR transcripts, the percentage of transcripts with APA-shift was unchanged (Figure 1B). In contrast, the extension of the 3′ UTR by 10% included longer non-3′ UTR sequences in the proximal region resulted in a reduced proportion of transcripts with APA-shift (Figure 1B). This is expected, as our library preparation was focused on short sequences starting from the poly(A) tail. This analysis shows that shifting the 3′ UTR split point around the middle of the 3′ UTR did not affect the percentage of transcripts with APA-shift on a transcriptome scale. Therefore, we continued with a split of the middle of the annotated 3′ UTR.

In the A17.1 most transcripts showed a shift to proximal (Figure 1C), agreeing with enhanced proximal APA in A17.1.[3,12] In a previous study, we used a difference protocol, using PAS-seq, to determine APA site utilization in A17.1.[3] The overlap between significant transcripts showing APA site utilization and transcripts with APA-shift was highly significant—77.3% of the APA-shift transcripts overlapped with PAS-seq (Figure 1D). We then also compared signal reads at the proximal and distal 3′ UTR regions in our study to the PAS-seq. We selected two transcripts for which PAS-seq was validated[3,25]: *Arih2* (ENSMUST00000013338) has two PAS at the 3′ UTR, and *Arih1* (ENSMUST00000171975) has multiple PAS at the 3′ UTR (Figure 1E). For both the localization of signal reads at proximal and distal regions was highly similar between the two procedures (Figure 1E). Thus, APA-shift can reproduce results obtained by a PAS-seq sequencing platform.

This confirms that our simple APA-shift protocol is valid to report changes in PAS utilization. We continued with the APA-shift analysis using the option where the 3′ UTR is divided in the middle into the proximal and distal regions.

To capture real-time changes in APA, we optimized the library preparation protocol and compared the standard protocol using 7 amplification cycles (called here 7C) to a protocol using 1 amplification cycle (called here 1C). As expected, the percentage of mapped reads was higher with the 7C protocol; however, the percentage of reads after UMI-based deduplication was higher with the 1C protocol (Table S2B; 64% ± 8.4%, and 84% ± 4.1%, respectively). The 1C protocol resulted in a library depth ~2-fold smaller than the 7C, but the average transcript size was 13% larger (Figure 2A). A principal-component analysis (PCA) showed similar differences between A17.1 and FVB samples (Figure S2A) in 7C and 1C protocols. However, with the 7C protocol, the average transcript size did not differ between FVB and A17.1, but with the 1C protocol the average transcript size was significantly smaller in A17.1 (Figure 2A). The same as with the 7C (standard) protocol also with the 1C protocol, an APA-shift to proximal was prominent (Figure 2B). Results from permuted half-split by shortening the extension of the 3′ UTR were similar between 7C and 1C (Figure S3). Since our APA-shift calculation depends on a split of the 3′ UTR in two, we expect a correlation with the 3′ UTR length, which was confirmed by a linear regression analysis (Figure S2D). Strikingly, a stronger correlation was found with the 1C (Figure S2D). Thus, the 1C protocol could be more beneficial for APA-shift calculation.

To further elucidate the differences between 1C and 7C protocols, we compared APA-shift directions. The proportion of transcripts with a shift to distal was significantly smaller in 1C compared with the 7C protocol (Figure 2C). Moreover, the overlap of transcripts between 1C and 7C was very high; the shift to proximal was 91%, but only 53% for the transcripts with a shift to distal (Figure 2D). To further investigate differences between 1C and 7C protocols, we focused on the prominent shift to proximal transcripts, defined as APA-shift >1 (log2), because some of the shift to distal transcripts could be false positives. The mean APA-shift value and the percentage of a prominent shift to proximal transcripts were higher in 1C compared to 7C (Figure 2E).

Because the A17.1 shows muscle pathology, we investigated whether the APA-shift of muscle transcripts differed between 7C and 1C. The proportion of muscle transcripts with 3′ UTR was higher in 1C; moreover, muscle transcripts with APA-shift were more abundant in the 1C protocol (Figure 2F). This indicates that the 1C protocol is beneficial for muscle transcripts. All of the subsequent analyses were carried out with the 1C dataset.

Reduced levels of *Pabpn1* isoforms correlate with APA-shift in A17.1

In A17.1 the expanded PABPN1 cDNA is overexpressed, suggesting that expression of the expanded PABPN1 leads to APA-shift. PABPN1 overexpression was confirmed by analysis of sequencing reads in the last exon (Figure 3A). However, sequencing reads at the 3′ UTR was slightly lower in the A17.1 (Figure 3A). The last exon is common between the endogenous Pabpn1 and the expanded PABPN1 transgene, but reads from the 3′ UTR are only from endogenous *Pabpn1* because the transgene lacks *Pabpn1* 3′ UTR. In mice, *Pabpn1* has eight annotated transcript isoforms, but five isoforms were found in the 1C dataset (Table S5). Levels of isoforms 201, 208, and 209 were significantly lower in A17.1 (Figure 3B), but expression levels of

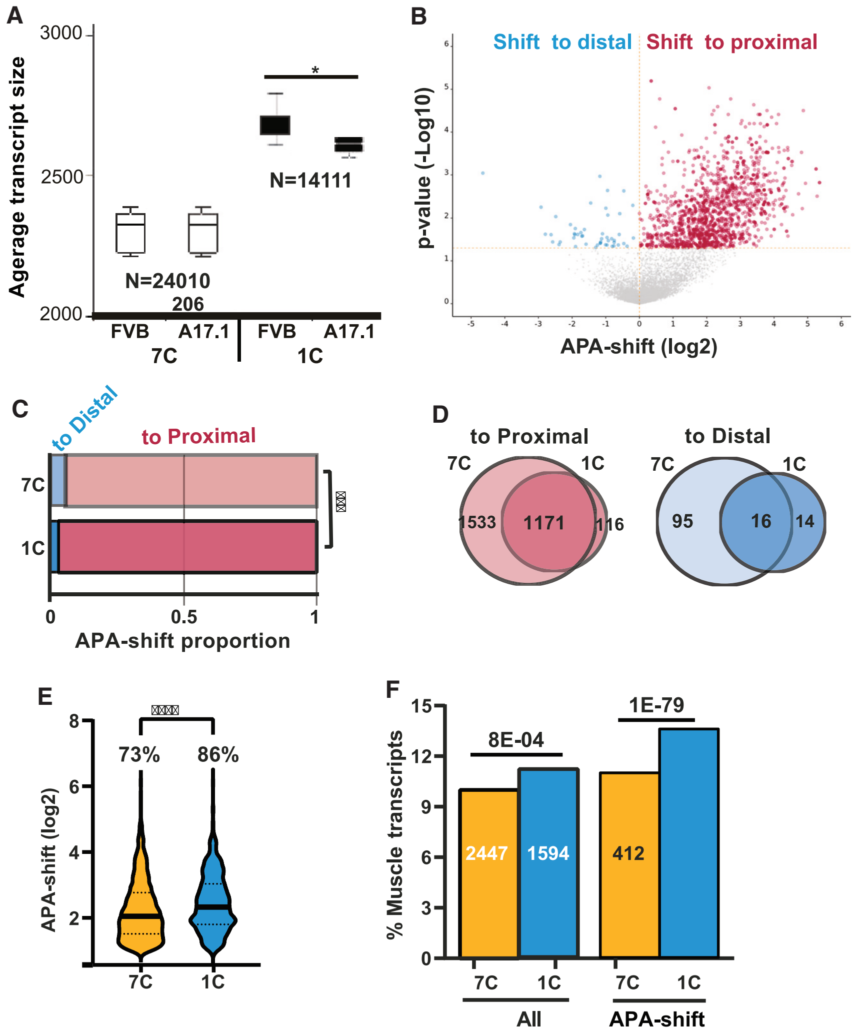

Figure 2. A comparison between 7C and 1C protocols and APA-shift results

(A) Boxplot shows average transcript size in 7C or 1C libraries in FVB and A17.1 muscles. Number of transcripts is depicted. Average is from N = 5. A significant difference between FVB and A17.1 assessed by the Student's t test (*$p < 0.05$).

(B) Volcano plot of APA-shift in A17.1 versus FVB mouse in the 1C RNA-seq. Mean log fold change and p values are from N = 5 per genotype. Transcripts with a shift to proximal are highlighted in red and a shift to distal in blue.

(C) Proportions of the significant APA-shift transcripts ($p < 0.05$ FDR) in 7C and 1C RNA-seq protocols. A shift to distal is depicted in blue and to proximal in red. Statistical difference was assessed with a chi-square test (***$p < 0.0001$).

(D) Venn diagrams show the overlap of the significant APA-shift transcripts ($p < 0.05$ FDR) between the 7C and the 1C datasets. A shift to proximal in is red and a shift to distal in blue.

(E) Violin plot shows APA-shift (log 2) of the most prominent APA-shift transcripts ($p < 0.05$ FDR and fold change >1) in 7C and 1C datasets. The percentage of most prominent transcripts from APA-shift transcripts is depicted. Statistical significance was assessed with ANOVA (****$p < 0.00001$).

(F) Bar chart shows the percentage of muscle genes encoded transcripts in the entire library (all transcripts) and the significant APA-shift transcripts in 7C and 1C protocols. The numbers of muscle transcripts is depicted in the bar. A statistical difference was assessed with a chi-square test and is depicted above the bars.

isoforms 202 and 207 were higher in A17.1 (Figure 3B; Table S5). The isoforms 201, 202, 207, and 208 encode for a protein containing RNA-binding motifs, but the isoform 208 lacks the first exon, containing the poly(alanine) tract (Figure 3C). The isoform 209 is a noncoding transcript. Considering the differences in expression levels of the five isoforms, we assessed a correlation between each isoform and APA-shift values. A negative correlation between isoforms 201, 208, and 209 expression levels and proximal APA-shift indicated that reduced levels in A17.1 correlate with APA-shift to proximal (Figure 3D). Over 70% of the transcripts with proximal APA-shift showed a significant correlation with isoforms 201 and 208, whereas the correlation with a shift to distal was lower (Figure 3D). In contrast to isoforms 201 and 208, a weaker positive correlation was found between isoform 207 and an APA-shift to proximal (Figure 3C). Isoform 202 showed a low correlation with an APA-shift to proximal (Figure 3D). Interestingly, reduced levels of isoform 209 showed the highest correlation with APA-shift, because over 90% of APA-shift transcripts showed a significant correlation (Figure 3E). The correlation between reduced levels of PABPN1 isoforms and APA-shift agrees with APA site utilization in PABPN1 knockout cells.[12] Together, our studies here suggest that reduced Pabpn1 level is the cause for APA-shift in A17.1. However, with this model we could not resolve the question of whether the expanded PABPN1 regulates APA-shifts the same as the normal PABPN1.

Low expression levels of expanded PABPN1 does not induce APA-shift

To investigate whether expression of the expanded PABPN1 causes APA-shift, we used a previously reported mouse muscle cell model for OPMD.[17] In this cell model, the expanded PABPN1 (A17) and the normal allele (A10) were overexpressed at similar levels in differentiated muscle cells.[17] PABPN1 overexpression was at low levels, driving only limited aggregation in myonuclei.[17] Therefore, this cell model is a relevant model to assess the effect of the expanded PABPN1 on PABPN1 activity, independent of its aggregation. Using the 1C protocol on RNA from A10 and A17, similar expression levels of PABPN1 transgene alleles were found (Figure 4A). In contrast to the A17.1 mouse model, APA-shift was not genome-wide, and a

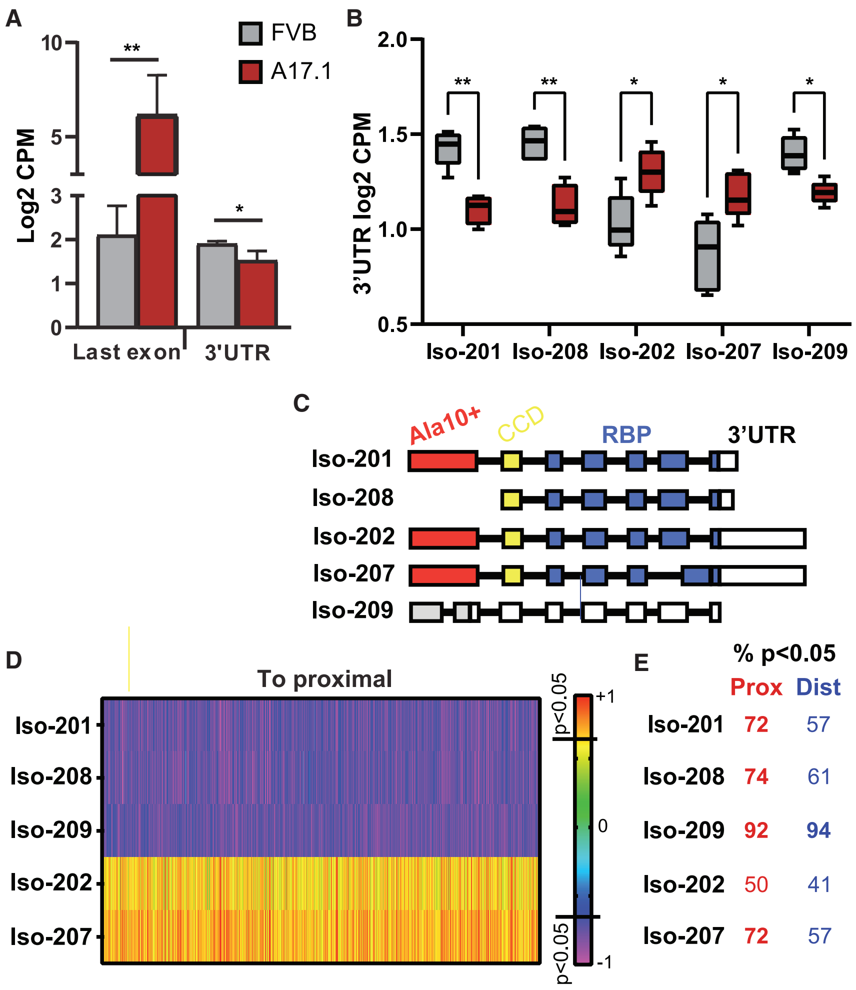

Figure 3. *Pabpn1* transcript levels in the A17.1 mouse model are associated with APA-shift

(A) Bar charts show *Pabpn1* levels measured from 1C RNA-seq last axon in counts per million (CPM), representing both endogenous Pabpn1 and the transgene Ala17 PABPN1. Reads counts from the 3′ UTR (sum of reads in all isoforms) represent the endogenous *Pabpn1*. Statistical difference was assessed with a Student's t test.

(B) Bar chart shows levels (CPM) of *Pabpn1* transcript isoforms in FVB and A17.1. Statistical difference was assessed with ANOVA, adjusted to multiple correction.

(C) A schematic presentation of *Pabpn1* isoforms, the first exon encoding for poly(alanine) is depicted in red, exon encoding for the coil-coil domain is in yellow, and the exons encoding for the RNA-binding domain are in blue. The 3′ UTR is depicted in white. Isoform 209 is annotated as nonsense mediated decay.

(D) Heatmap of Spearman correlation (r) between APA-shift ratio (log 2) in the APA-shift significant transcripts and Pabpn1 isoforms (Iso) expression levels (log2). Correlation analysis was carried out for transcripts with APA-shift to proximal or to distal separately, for all 5 Pabpn1 isoforms.

(E) The percentage of transcripts with significant correlation ($p < 0.05$) is depicted at the right of the heatmap.

prominent shift to proximal was not found in A17 cells (Figure 4B). To validate these results, we repeated the entire procedure using the 7C protocol, which resulted in similar low APA-shift as in C1 (Figure S4). Moreover, with both 1C and 7C protocols, the expression of A17 did not lead to a significant APA-shift (Figure S4). This demonstrates that in the A17 cell model, APA is not activated genome-wide.

Interestingly, the expression of the expanded PABPN1 did not cause a reduction in endogenous Pabpn1 expression levels (Figure 4A). Isoforms 201 and 208 showed higher levels in A17, but the fold change was small (1.7-fold) (Figure 4C). A correlation between isoforms 201 or 208 expression levels and APA-shift ($p < 0.05$, no FDR) was minimal, with only 10% of transcripts with APA-shift showing a significant correlation (Figure 4D). In contrast to the results in A17.1, isoform 209 did not correlate with APA-shift (Figure 4D).

Since APA-shift in muscle transcripts was prominent in A17.1 tibialis anterior muscle, we assessed whether in the cell model muscle transcripts were enriched among the APA-shift ($p < 0.05$, no FDR). The percentage of muscle transcripts in the 1C dataset was similar in mice and muscle cell lines (Figure 4E). However, in the A17 muscle cell culture, the proportion of muscle transcripts with APA-shift was significantly lower than the proportion of muscle transcripts in the entire dataset (Figure 4E). In agreement with little similarities between A17.1 and A17 effect on APA-shift, an overlap between A17 and A17.1 of transcripts with APA-shift was insignificant (Figure 4F). Together, the results here indicate that expression of the expanded PABPN1 does not cause APA-shift.

APA-shift in OPMD is enriched in muscle-associated pathways

Thus far, APA as a readout for PABPN1 activity was investigated in OPMD models, but not in OPMD individuals. We applied the 1C protocol with the APA-shift analysis on RNA from vastus lateralis muscle of OPMD (N = 10) and control (N = 8) subjects. To our surprise, significant APA-shift ($p < 0.05$, FDR) was not massive in OPMD, and a prominent shift to proximal was not found (Figure 5A). Visualization of reads peak at the 3′ UTR, agreeing with genomic localization of reads peak in using human samples PAS-seq or the poly(A) database (Figure S4). A PCA of APA-shift values in A17.1 showed a clear separation between control and OPMD models, whereas in humans and the cell model, a separation based on genotype was not found (Figure S4). Despite the low number of transcripts with a significant APA-shift in OPMD, enrichment analysis showed an exclusive enrichment for myogenesis (Figure 5B), with most of the muscle transcripts showing

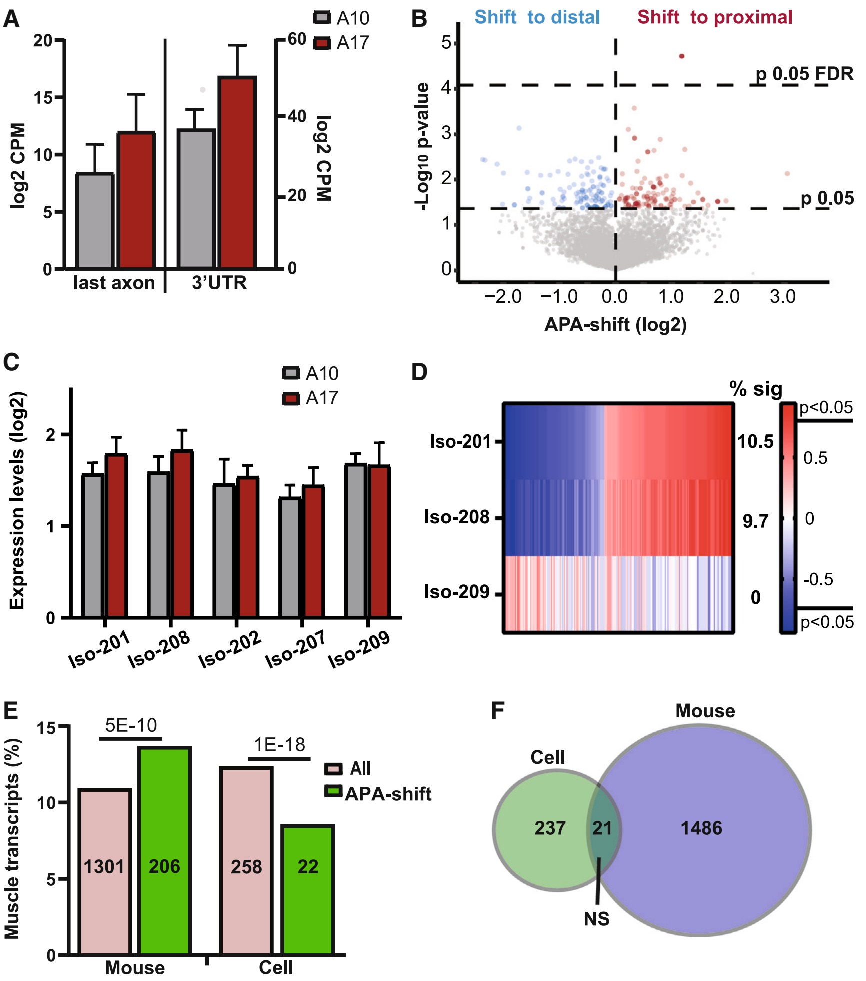

Figure 4. APA-shift in differentiated (fused) A17 muscle cell model is not associated with PABPN1 levels

(A) Bar charts show *Pabpn1* levels measured from 1C RNA-seq last axon, representing both endogenous Pabpn1 and the transgenes Ala17 or Ala10 PABPN1. Read counts (CPM) from the 3′ UTR (sum of reads in all isoforms) represent the endogenous *Pabpn1*. Statistical difference was assessed with a Student's t test.

(B) Volcano plot of APA-shift in A17 versus A10 muscle cells in the 1C RNA-seq. Mean log fold change and p values are from N = 3 per cell line. Dashed horizontal lines denote 0.05 or 0.05 FDR p value cutoff.

(C) Bar chart shows levels of *Pabpn1* transcripts isoform expression levels in A10 and A17 muscle cell model. Read counts are from the 3′ UTR. Statistical analysis was assessed with ANOVA, and did not show statistical difference.

(D) Heatmap of Spearman correlation (r) between APA-shift ratio (log 2) in the APA-shift transcripts ($p < 0.05$) and Pabpn1 isoforms (Iso) expression levels (log2). Correlation analysis was carried out on transcripts with significant APA-shift for 3 Pabpn1 isoforms. The percentage of transcripts with significant correlation ($p < 0.05$) is depicted at the right of the heatmap.

(E) Bar chart shows the percentage of the transcripts encoding for muscle genes in the entire library (all transcripts) and the APA-shift transcripts in the OPMD mouse and cell datasets. Numbers of transcripts are depicted in each bar. Statistical difference was assessed with a chi-square test.

(F) Venn diagram shows transcript overlap for APA-shift transcripts in cell and mouse OPMD models. Statistical significance of overlap was assessed with a chi-square test. NS, not significant.

a shift to proximal (Table S5). The overlap of muscle genes with APA-shift between OPMD and A17.1 was significant, and most of the genes showed an APA-shift to proximal (Figure 5C). The gene list is depicted in Figure 5A. The similarity between OPMD and A17.1 APA-shift was also found in GO enrichment analysis. Similar terms related to muscle pathology were enriched in OPMD and A17.1 (Figure 5D).

In a previous study, we showed reduced *PABPN1* levels in OPMD.[13] Here, we investigated whether *PABPN1* isoforms may affect the APA-shift in OPMD. In humans, the three isoforms 201 (ENST00000216727), 202 (ENST00000397276), and 207 (ENST00000556821) are the most abundant in skeletal muscles (https://www.proteinatlas.org/ and Uhlén et al.[26]). The isoforms 201 and 002 encode for the full-length PABPN1 but differ in UTR sequences (Figure 5E). Isoform 207 lacks the majority of the first exon, including the poly(alanine) expansion (Figure 5E). Consistent with our previous study, also in this study we detected reduced *PABPN1* levels in OPMD. Using primers specific to isoform 201 or isoform 207, reduced PABPN1 levels was found for isoform 207 (Figure 5F). This suggests that APA-shift in OPMD is driven by reduced isoform 207 levels.

Discussion

Here, we describe a procedure that assesses APA-shift in an optimized library preparation protocol, and we demonstrate that this procedure robustly reports APA-shift in OPMD. Genome-wide alterations in transcripts expression levels due to APA have been linked to a range of pathological conditions.[8] Cell types can be distinguished by patterns of APA marking cell specificity.[10] Also in pathological conditions, APA has been found in a wide range of tissues, including different cancer types,[27–30] heart,[31] and brain[32] cells of the immune response.[33] Here, we show APA-shift in skeletal muscles from OPMD individuals. Thus, our study corroborates other studies suggesting that APA is an important regulatory layer in human pathologies.

Identification of APA site utilization requires specific RNA-seq protocols, such as PAS-seq, which were instrumental in distinguishing between the proximal and distal PASs.[3,34] Proximal PASs represent APA sites in the A17.1 OPMD mouse model.[3,12] However, PAS-seq is costly and hence less suitable for patient material. Computational pipelines were developed to accurately identify alteration

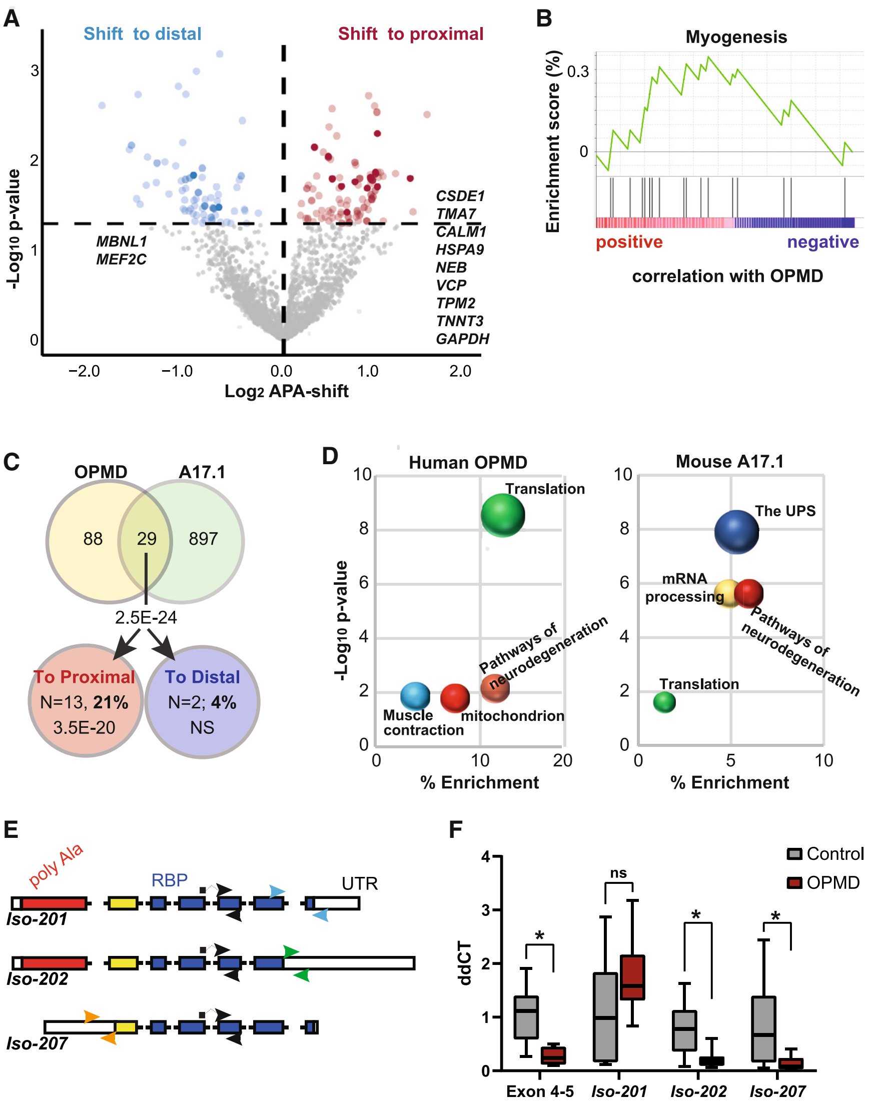

Figure 5. APA shift in OPMD muscles
(A) Volcano plot of APA-shift in subjects with OPMD versus control's vastus lateralis in 1C RNA-seq. Mean log fold change and p values are from N = 10 and 8 per genotype, respectively. Significant transcripts ($p < 0.05$ FDR) with a switch to proximal are highlighted in red and a switch to distal in blue. Genes overlapping with A17.1 are highlighted in dark red or dark blue, and gene names are depicted.
(B) Enrichment analysis of OPMD APA-shift transcripts. Visualization of GSEA enrichment. Myogenesis was found significant, and most transcripts showed positive correlation with OPMD.
(C) Venn diagram shows the overlap of genes with significant APA-shift between OPMD and A17.1 in yellow and green, respectively, and among genes with proximal (red) or distal (blue) APA-shift. The overlap significance was assessed with a chi-square test. OPMD APA-shift genes overlapping with A17.1 and enriched in skeletal muscles are depicted.
(D) Dot plot visualization of functional term enrichment for the proximal APA-shift genes in OPMD and in A17.1. Redundancy was removed using annotation clustering; the most significant term within an enrichment cluster is depicted. Similar terms are marked with the same color.
(E) A schematic presentation of *PABPN1* isoforms 201, 202, and 207. Exons are depicted in full boxes and introns in dashed lines. The first exon encoding for poly(alanine) is depicted in red, exon encoding for the coil-coil domain is in yellow, and the exons encoding for the RNA-binding domain are in blue. UTR regions are depicted in white. Primer sets that were used for qRT-PCR are denoted with arrows: exon 4–5 in black arrows; isoform 001 in blue; isoform 002 in green, and isoform 004 in orange.
(F) Bar chart shows normalized ddCt values of *PABPN1* isoforms in control and OPMD. Statistical difference was assessed with ANOVA; $*p < 0.05$; $**p < 0.0005$.

in APA and PAS site utilization.[35–37] Since APA is cell-type or species- specific,[10] application of this procedure in less studied cells, such as muscle cells, could be tricky.[6] The C1 protocol in combination with an APA-shift computational pipeline reports PABPN1 function as a quantitative readout. Compared to the standard protocol, in the 1C protocol, RNA dynamics are closer to the *in vivo* situation. We show that the 1C protocol is more suitable for muscle transcripts and detecting APA-shifts. However, the 1C sequencing depth is limited and not as deep as the standard protocol, and is more dependent on a similar amount and quality of RNA input across samples. The latter is a specific limitation for human tissues that are stored for a long time. We also acknowledge the limitation of the APA-shift pipeline; since it is independent of PAS genomic information it provides an estimation for PAS utilization. However, our results with APA-shift in the mouse OPMD model showed nearly 80% transcript overlap to our previous independent study, demonstrating APA-shift reliability. Despite these limitations, the APA-shift pipeline is simple and can be applied to any organism without prior knowledge of PAS or APA sequence. This pipeline results in a single value for significant APA-shift direction and can be applied to existing RNA-seq data.

The functional consequences of APA at the 3′ UTR are multilayered: (1) APA resulting in shorter 3′ UTR leads to higher mRNA stability[3,12]; (2) transcripts with longer 3′ UTR seem to retain in the nucleus as compared to transcripts with shorter 3′ UTR[38]; (3) translation efficiency of transcripts with APA at the 3′ UTR is lower compared to transcripts with long 3′UTR. In agreement with higher nuclear export of short transcripts, we recently showed that transcripts with APA at the 3′ UTR show higher abundance in heavy polysomes.[6]

Although APA site utilization is regulated by multiple nuclear RNA-binding proteins, reduced PABPN1 levels

have been implicated in APA site utilization in different cancers.[30,39] Reduced PABPN1 levels lead to muscle wasting,[40,41] and have been also found in OPMD individuals.[13,42] Although genome-wide APA has been previously demonstrated in a mouse model for OPMD that overexpresses the expanded PABPN1,[3,12] in OPMD muscles we found APA-shift mainly in muscle transcripts. The limited APA-shift we found in OPMD could be attributed to the C1 protocol that was not genome-wide in RNA from human muscles. Also, in lung cancer cells, APA was found in a small number of genes regulating the cell cycle.[39] Together, it is possible that APA is initially found in a small subset of transcripts that may drive cell damage. In skeletal muscles, the muscle transcripts with APA-shift could contribute to muscle weakness and atrophy.

Our results here show that the expression of the pathogenic, alanine-expanded PABPN1 does not cause APA-shift. Instead, reduced expression levels of a subset of PABPN1 isoforms correlate with APA-shift in both the A17.1 mouse model and OPMD. It is possible that (over) expression of the expanded PABPN1 leads to reduced expression of the normal transcript isoforms. It was previously suggested by others and us that PABPN1 aggregation leads reduced levels of the functional PABPN1,[43,44] but the exact molecular mechanism is unknown. In the mouse, the noncoding *Pabpn1* isoform 209 was expressed at levels close to the protein-coding isoforms and showed the strongest correlation with APA-shift. Noncoding RNAs can regulate RNA expression levels.[45] A model for the PABPN1 self-regulation mechanism has been proposed, in which high PABPN1 protein expression levels lead to reduced expression via mRNA decay.[46] The human noncoding PABPN1 isoforms are not abundant in skeletal muscles (https://www.proteinatlas.org). OPMD is mostly considered a gain-of-function disease due to the presence of nuclear aggregates.[47] Our study suggests that PABPN1 loss of function also contributes to OPMD. Interestingly, we identify that levels of the isoform lacking the alanine expansion region are reduced in OPMD. The role of the truncated isoform is unknown, but potentially it could open specific therapeutical options for OPMD.

Conclusion

The combination of 1C protocol and APA-shift quantification allowed us to identify and report APA-shift in OPMD individuals. APA-shift in OPMD is predominantly enriched in muscle genes and genes affecting neurodegeneration pathways. The mutated PABPN1 does not affect APA-shift, suggesting that its expression does not compromise PABPN1 activity. Instead, lower expression of PABPN1 isoforms seems to compromise PABPN1 activity that in skeletal muscles muscle transcripts are initially affected. Considering the involvement of APA in a broad range of human pathologies, investigating the potential role of RNA-binding proteins regulating APA in muscle transcripts could offer valuable insights into the molecular mechanisms underlying muscle wasting, not only in OPMD but also in other related pathologies.

Data and code availability

APA-shift https://github.com/lumc-sasc/pub-PABPN1_APA-shift_Raz.

The datasets generated in this study are available upon request from the lead author.

Supplemental information

Supplemental information can be found online at https://doi.org/10.1016/j.xhgg.2024.100269.

Acknowledgments

We thank Peter A.C. ‘t Hoen (Department of Medical BioSciences, Radboud University Medical Center, Nijmegen, the Netherlands) for critical comments and useful suggestions on the manuscript. This study is supported by the Human Genetics NGS funding and by the kind support of argenx. The study funders have no role in study design and data collection, and both agreed with the publication.

Author contributions

RNA isolation, bioinformatics analysis, and qRT-PCR: M.S. Bioinformatics and design of APA-shift calculation: H.M. Development of RNA-seq protocols: Y.A. and S.K. Study design, supervision, obtained funding acquisition: V.R. All of the authors contributed to writing the manuscript.

Declaration of interests

(1) The procedures followed were in accordance with the ethical standards of the responsible committee on human experimentation (Radboud medical ethics board) and proper informed consent was obtained from all of the subjects.
(2) Competing interests: All of the authors declare no competing interests. The study funders have no competing interest in this study.
(3) Consent for publication: Individuals’ data are not shared. Informed consent specifies the use of biopsies for research and publication.
(4) Availability of data and materials: APA-shift results in all models and in OPMD are provided in Table S3. The raw datasets and codes supporting the present study have not been deposited in a public repository because human data cannot be shared, but they are available from the corresponding author on request.

Received: August 29, 2023
Accepted: January 5, 2024

Web resources

NCBI Primer design tool, https://www.ncbi.nlm.nih.gov/tools/primer-blast/

Tissue specificity, https://www.proteinatlas.org.

GSEA enrichment, https://www.gsea-msigdb.org/gsea/index.jsp.

GO database, www.geneontology.org.

Ultraplex, https://github.com/ulelab/ultraplex

IGV, https://igv.org/doc/desktop/

DAVID bioinformatics, https://david.ncifcrf.gov/

ggvolcanoR, https://ggvolcanor.erc.monash.edu/

A catalog of human genes and genetic disorders, https://www.omim.org/

References

1. Ren, F., Zhang, N., Zhang, L., Miller, E., and Pu, J.J. (2020). Alternative Polyadenylation: a new frontier in post transcriptional regulation. Biomark. Res. *8*, 67.
2. Andreassi, C., and Riccio, A. (2009). To localize or not to localize: mRNA fate is in 3′UTR ends. Trends Cell Biol. *19*, 465–474.
3. de Klerk, E., Venema, A., Anvar, S.Y., Goeman, J.J., Hu, O., Trollet, C., Dickson, G., den Dunnen, J.T., van der Maarel, S.M., Raz, V., and 't Hoen, P.A.C. (2012). Poly(A) binding protein nuclear 1 levels affect alternative polyadenylation. Nucleic Acids Res. *40*, 9089–9101.
4. Mitschka, S., and Mayr, C. (2022). Context-specific regulation and function of mRNA alternative polyadenylation. Nat. Rev. Mol. Cell Biol. *23*, 779–796.
5. Tanguay, R.L., and Gallie, D.R. (1996). Translational efficiency is regulated by the length of the 3′ untranslated region. Mol. Cell Biol. *16*, 146–156.
6. Mei, H., Boom, J., El Abdellaoui, S., Abdelmohsen, K., Munk, R., Martindale, J.L., Kloet, S., Kielbasa, S.M., Sharp, T.H., Gorospe, M., and Raz, V. (2022). Alternative Polyadenylation Utilization Results in Ribosome Assembly and mRNA Translation Deficiencies in a Model for Muscle Aging. J. Gerontol. A Biol. Sci. Med. Sci. *77*, 1130–1140.
7. Tian, B., and Manley, J.L. (2017). Alternative polyadenylation of mRNA precursors. Nat. Rev. Mol. Cell Biol. *18*, 18–30.
8. Gruber, A.J., and Zavolan, M. (2019). Alternative cleavage and polyadenylation in health and disease. Nat. Rev. Genet. *20*, 599–614.
9. Nishtala, S., Neelamraju, Y., and Janga, S.C. (2016). Dissecting the expression relationships between RNA-binding proteins and their cognate targets in eukaryotic post-transcriptional regulatory networks. Sci. Rep. *6*, 25711.
10. Shulman, E.D., and Elkon, R. (2019). Cell-type-specific analysis of alternative polyadenylation using single-cell transcriptomics data. Nucleic Acids Res. *47*, 10027–10039.
11. Chang, J.W., Yeh, H.S., and Yong, J. (2017). Alternative Polyadenylation in Human Diseases. Endocrinol. Metab. *32*, 413–421.
12. Jenal, M., Elkon, R., Loayza-Puch, F., van Haaften, G., Kühn, U., Menzies, F.M., Oude Vrielink, J.A.F., Bos, A.J., Drost, J., Rooijers, K., et al. (2012). The poly(A)-binding protein nuclear 1 suppresses alternative cleavage and polyadenylation sites. Cell *149*, 538–553.
13. Anvar, S.Y., Raz, Y., Verway, N., van der Sluijs, B., Venema, A., Goeman, J.J., Vissing, J., van der Maarel, S.M., 't Hoen, P.A.C., van Engelen, B.G.M., and Raz, V. (2013). A decline in PABPN1 induces progressive muscle weakness in oculopharyngeal muscle dystrophy and in muscle aging. Aging (Albany NY) *5*, 412–426.
14. Brais, B., Bouchard, J.P., Xie, Y.G., Rochefort, D.L., Chrétien, N., Tomé, F.M., Lafrenière, R.G., Rommens, J.M., Uyama, E., Nohira, O., et al. (1998). Short GCG expansions in the PABP2 gene cause oculopharyngeal muscular dystrophy. Nat. Genet. *18*, 164–167.
15. Tavanez, J.P., Calado, P., Braga, J., Lafarga, M., and Carmo-Fonseca, M. (2005). In vivo aggregation properties of the nuclear poly(A)-binding protein PABPN1. Rna *11*, 752–762.
16. Trollet, C., Anvar, S.Y., Venema, A., Hargreaves, I.P., Foster, K., Vignaud, A., Ferry, A., Negroni, E., Hourde, C., Baraibar, M.A., et al. (2010). Molecular and phenotypic characterization of a mouse model of oculopharyngeal muscular dystrophy reveals severe muscular atrophy restricted to fast glycolytic fibres. Hum. Mol. Genet. *19*, 2191–2207.
17. Raz, V., Routledge, S., Venema, A., Buijze, H., van der Wal, E., Anvar, S., Straasheijm, K.R., Klooster, R., Antoniou, M., and van der Maarel, S.M. (2011). Modeling oculopharyngeal muscular dystrophy in myotube cultures reveals reduced accumulation of soluble mutant PABPN1 protein. Am. J. Pathol. *179*, 1988–2000.
18. de Leeuw, R.H., Garnier, D., Kroon, R.M.J.M., Horlings, C.G.C., de Meijer, E., Buermans, H., van Engelen, B.G.M., de Knijff, P., and Raz, V. (2019). Diagnostics of short tandem repeat expansion variants using massively parallel sequencing and componential tools. Eur. J. Hum. Genet. *27*, 400–407.
19. Bagnoli, J.W., Ziegenhain, C., Janjic, A., Wange, L.E., Vieth, B., Parekh, S., Geuder, J., Hellmann, I., and Enard, W. (2018). Sensitive and powerful single-cell RNA sequencing using mcSCRB-seq. Nat. Commun. *9*, 2937.
20. Datlinger, P., Rendeiro, A.F., Boenke, T., Senekowitsch, M., Krausgruber, T., Barreca, D., and Bock, C. (2021). Ultra-high-throughput single-cell RNA sequencing and perturbation screening with combinatorial fluidic indexing. Nat. Methods *18*, 635–642.
21. Wilkins, O.G., Capitanchik, C., Luscombe, N.M., and Ule, J. (2021). Ultraplex: A rapid, flexible, all-in-one fastq demultiplexer. Wellcome Open Res. *6*, 141.
22. Mullan, K.A., Bramberger, L.M., Munday, P.R., Goncalves, G., Revote, J., Mifsud, N.A., Illing, P.T., Anderson, A., Kwan, P., Purcell, A.W., and Li, C. (2021). ggVolcanoR: A Shiny app for customizable visualization of differential expression datasets. Comput. Struct. Biotechnol. J. *19*, 5735–5740.
23. Subramanian, A., Tamayo, P., Mootha, V.K., Mukherjee, S., Ebert, B.L., Gillette, M.A., Paulovich, A., Pomeroy, S.L., Golub, T.R., Lander, E.S., and Mesirov, J.P. (2005). Gene set enrichment analysis: a knowledge-based approach for interpreting genome-wide expression profiles. Proc. Natl. Acad. Sci. USA *102*, 15545–15550.
24. Huang, D.W., Sherman, B.T., Tan, Q., Kir, J., Liu, D., Bryant, D., Guo, Y., Stephens, R., Baseler, M.W., Lane, H.C., and Lempicki, R.A. (2007). DAVID Bioinformatics Resources: expanded annotation database and novel algorithms to better extract biology from large gene lists. Nucleic Acids Res. *35*, W169–W175.
25. Raz, V., Buijze, H., Raz, Y., Verwey, N., Anvar, S.Y., Aartsma-Rus, A., and van der Maarel, S.M. (2014). A novel feed-forward loop between ARIH2 E3-ligase and PABPN1 regulates aging-

associated muscle degeneration. Am. J. Pathol. *184*, 1119–1131.

26. Uhlén, M., Fagerberg, L., Hallström, B.M., Lindskog, C., Oksvold, P., Mardinoglu, A., Sivertsson, Å., Kampf, C., Sjöstedt, E., Asplund, A., et al. (2015). Proteomics. Tissue-based map of the human proteome. Science *347*, 1260419.
27. Yuan, F., Hankey, W., Wagner, E.J., Li, W., and Wang, Q. (2021). Alternative polyadenylation of mRNA and its role in cancer. Genes Dis. *8*, 61–72.
28. Mayr, C., and Bartel, D.P. (2009). Widespread shortening of 3′UTRs by alternative cleavage and polyadenylation activates oncogenes in cancer cells. Cell *138*, 673–684.
29. Goering, R., Engel, K.L., Gillen, A.E., Fong, N., Bentley, D.L., and Taliaferro, J.M. (2021). LABRAT reveals association of alternative polyadenylation with transcript localization, RNA binding protein expression, transcription speed, and cancer survival. BMC Genom. *22*, 476.
30. Chen, L., Dong, W., Zhou, M., Yang, C., Xiong, M., Kazobinka, G., Chen, Z., Xing, Y., and Hou, T. (2023). PABPN1 regulates mRNA alternative polyadenylation to inhibit bladder cancer progression. Cell Biosci. *13*, 45.
31. Creemers, E.E., Bawazeer, A., Ugalde, A.P., van Deutekom, H.W.M., van der Made, I., de Groot, N.E., Adriaens, M.E., Cook, S.A., Bezzina, C.R., Hubner, N., et al. (2016). Genome-Wide Polyadenylation Maps Reveal Dynamic mRNA 3′-End Formation in the Failing Human Heart. Circ. Res. *118*, 433–438.
32. Cui, Y., Arnold, F.J., Peng, F., Wang, D., Li, J.S., Michels, S., Wagner, E.J., La Spada, A.R., and Li, W. (2023). Alternative polyadenylation transcriptome-wide association study identifies APA-linked susceptibility genes in brain disorders. Nat. Commun. *14*, 583.
33. Jia, X., Yuan, S., Wang, Y., Fu, Y., Ge, Y., Ge, Y., Lan, X., Feng, Y., Qiu, F., Li, P., et al. (2017). The role of alternative polyadenylation in the antiviral innate immune response. Nat. Commun. *8*, 14605.
34. Yoon, Y., Soles, L.V., and Shi, Y. (2021). PAS-seq 2: A fast and sensitive method for global profiling of polyadenylated RNAs. Methods Enzymol. *655*, 25–35.
35. Ye, C., Long, Y., Ji, G., Li, Q.Q., and Wu, X. (2018). APAtrap: identification and quantification of alternative polyadenylation sites from RNA-seq data. Bioinformatics *34*, 1841–1849.
36. Ha, K.C.H., Blencowe, B.J., and Morris, Q. (2018). QAPA: a new method for the systematic analysis of alternative polyadenylation from RNA-seq data. Genome Biol. *19*, 45.
37. Long, Y., Zhang, B., Tian, S., Chan, J.J., Zhou, J., Li, Z., Li, Y., An, Z., Liao, X., Wang, Y., et al. (2023). Accurate transcriptome-wide identification and quantification of alternative polyadenylation from RNA-seq data with APAIQ. Genome Res. *33*, 644–657.
38. Tang, P., Yang, Y., Li, G., Huang, L., Wen, M., Ruan, W., Guo, X., Zhang, C., Zuo, X., Luo, D., et al. (2022). Alternative polyadenylation by sequential activation of distal and proximal PolyA sites. Nat. Struct. Mol. Biol. *29*, 21–31.
39. Ichinose, J., Watanabe, K., Sano, A., Nagase, T., Nakajima, J., Fukayama, M., Yatomi, Y., Ohishi, N., and Takai, D. (2014). Alternative polyadenylation is associated with lower expression of PABPN1 and poor prognosis in non-small cell lung cancer. Cancer Sci. *105*, 1135–1141.
40. Riaz, M., Raz, Y., van Putten, M., Paniagua-Soriano, G., Krom, Y.D., Florea, B.I., and Raz, V. (2016). PABPN1-Dependent mRNA Processing Induces Muscle Wasting. PLoS Genet. *12*, e1006031.
41. Olie, C.S., Riaz, M., Konietzny, R., Charles, P.D., Pinto-Fernandez, A., Kiełbasa, S.M., Aartsma-Rus, A., Goeman, J.J., Kessler, B.M., and Raz, V. (2019). Deacetylation Inhibition Reverses PABPN1-Dependent Muscle Wasting. iScience *12*, 318–332.
42. Roth, F., Dhiab, J., Boulinguiez, A., Mouigni, H.-R., Lassche, S., Negroni, E., Muraine, L., Marhic, A., Oliver, A., Lainé, J., et al. (2022). Assessment of PABPN1 nuclear inclusions on a large cohort of patients and in a human xenograft model of oculopharyngeal muscular dystrophy. Acta Neuropathol. *144*, 1157–1170.
43. Raz, Y., and Raz, V. (2014). Oculopharyngeal muscular dystrophy as a paradigm for muscle aging. Front. Aging Neurosci. *6*, 317.
44. Banerjee, A., Apponi, L.H., Pavlath, G.K., and Corbett, A.H. (2013). PABPN1: molecular function and muscle disease. FEBS J. *280*, 4230–4250.
45. Statello, L., Guo, C.-J., Chen, L.-L., and Huarte, M. (2021). Gene regulation by long non-coding RNAs and its biological functions. Nat. Rev. Mol. Cell Biol. *22*, 96–118.
46. Bergeron, D., Pal, G., Beaulieu, Y.B., Chabot, B., and Bachand, F. (2015). Regulated Intron Retention and Nuclear Pre-mRNA Decay Contribute to PABPN1 Autoregulation. Mol. Cell Biol. *35*, 2503–2517.
47. Malerba, A., Klein, P., Bachtarzi, H., Jarmin, S.A., Cordova, G., Ferry, A., Strings, V., Espinoza, M.P., Mamchaoui, K., Blumen, S.C., et al. (2017). PABPN1 gene therapy for oculopharyngeal muscular dystrophy. Nat. Commun. *8*, 14848.

HGGA, Volume *5*

Supplemental information

PABPN1 loss-of-function causes APA-shift in oculopharyngeal muscular dystrophy

Milad Shademan, Hailiang Mei, Baziel van Engelen, Yavuz Ariyurek, Susan Kloet, and Vered Raz

Supplementary data

A. Supplementary Tables

Table S1: Gender and age information of OPMD and control subjects whose RNA from vastus lateralis muscle was used in this study.

OPMD			Control		
Sample nr	**Age range**	**Gender**	Sample nr	**Age range**	**Gender**
1	60-69	f	3	20-29	m
2	40-49	m	4	60-69	m
3	50-59	m	5	60-69	f
4	80-89	f	6	80-89	f
5	40-49	f	7	30-39	m
6	50-59	f	8	40-49	f
7	40-49	f	11	80-49	f
8	70-79	m	12	50-59	m
9	60-69	f			
10	40-49	f			

Table S2: Data sets description

**A. Experiment**	**Sample nr.**	**Library prep**	**Transcript exclusion criteria**	**Transcript nr. (3'UTR)**	**Included transcript nr.**	**Transcript with APA shift**
**Mouse (FVB and A17.1)**	5 + 5	7C	∑ reads proximal =<10 and	52218	24106	3728
	5 + 5	1C			14111	1507
**Mouse cell line (A10 and A17)**	3 + 3	7C			23869	753
	3 + 3	1C			8278	258
**Human (control and OPMD)**	8 + 10	1C	Reads at proximal region in = > 30% of samples	85619	3113	215

**B.**	**Category**	**Total input reads**	**Unmapped reads**		**Mapped reads**		**Reads after UMI based dedup (transcriptome based approach)**	
**7C**	FVB_Q2	12404400	337395	2.72%	12067005	97.28%	6939372	57.51%
	FVB_Q3	13815810	342575	2.48%	13473235	97.52%	7080216	52.55%
	FVB_Q4	12525017	301601	2.41%	12223416	97.59%	8004434	65.48%
	FVB_Q5	13073935	320279	2.45%	12753656	97.55%	7985539	62.61%
	FVB_Q6	11424354	297712	2.61%	11126642	97.39%	7445899	66.92%
	A17.1_Q2	11229458	315988	2.81%	10913470	97.19%	6485672	59.43%
	A17.1_Q3	10951353	310997	2.84%	10640356	97.16%	5586852	52.51%
	A17.1_Q4	11602273	334719	2.88%	11267554	97.12%	6286475	55.79%
	A17.1_Q5	10327784	294344	2.85%	10033440	97.15%	5854875	58.35%
	A17.1_Q6	10357987	319769	3.09%	10038218	96.91%	6209008	61.85%
	A10_1	14786997	514065	3.48%	14272932	96.52%	10443091	73.17%

	A10_2	9284162	321864	3.47%	8962298	96.53%	6911140	77.11%
	A10_3	9044039	292607	3.24%	8751432	96.76%	6568761	75.06%
	A17_1	10643513	388054	3.65%	10255459	96.35%	7073487	68.97%
	A17_2	11148411	370948	3.33%	10777463	96.67%	8018058	74.40%
	A17_3	11290889	341323	3.02%	10949566	96.98%	7653099	69.89%
**1C**	FVB_Q2	1550669	475119	30.64%	1075550	69.36%	871531	81.03%
	FVB_Q3	1816421	546595	30.09%	1269826	69.91%	960845	75.67%
	FVB_Q4	1635290	494250	30.22%	1141040	69.78%	982341	86.09%
	FVB_Q5	2274844	696713	30.63%	1578131	69.37%	1316496	83.42%
	FVB_Q6	2268734	698919	30.81%	1569815	69.19%	1344212	85.63%
	A17.1_Q2	1418780	443039	31.23%	975741	68.77%	791623	81.13%
	A17.1_Q3	932978	289091	30.99%	643887	69.01%	498184	77.37%
	A17.1_Q4	1606702	483546	30.10%	1123156	69.90%	878087	78.18%
	A17.1_Q5	1716088	525516	30.62%	1190572	69.38%	941188	79.05%
	A17.1_Q6	1638100	507705	30.99%	1130395	69.01%	930914	82.35%
	A10_1	556663	146303	26.28%	410360	73.72%	364145	88.74%
	A10_2	484524	131897	27.22%	352627	72.78%	315090	89.36%
	A10_3	355590	104663	29.43%	250927	70.57%	223657	89.13%
	A17_1	153917	44902	29.17%	109015	70.83%	97100	89.07%
	A17_2	280383	78810	28.11%	201573	71.89%	179363	88.98%
	A17_3	530802	147575	27.80%	383227	72.20%	338299	88.28%
**Control subjects**	c11	2117733	1824795	86.17%	292938	13.83%	92274	31.50%
	c12	4182735	3391372	81.08%	791363	18.92%	236127	29.84%
	c3	1840311	1558919	84.71%	281392	15.29%	81990	29.14%
	c4	2267725	1903233	83.93%	364492	16.07%	84056	23.06%
	c5	1686682	1388817	82.34%	297865	17.66%	84010	28.20%
	c6	2198539	1861224	84.66%	337315	15.34%	126287	37.44%
	c7	1467447	1329313	90.59%	138134	9.41%	70051	50.71%
	c8	3574491	3171647	88.73%	402844	11.27%	179056	44.45%
**OPMD subjects**	p1	4236462	3618811	85.42%	617651	14.58%	179128	29.00%
	p2	3269827	2851317	87.20%	418510	12.80%	140022	33.46%
	p3	2399867	2073306	86.39%	326561	13.61%	101192	30.99%
	p4	2868232	2414007	84.16%	454225	15.84%	124061	27.31%
	p5	3371018	2689161	79.77%	681857	20.23%	176315	25.86%
	p6	1674483	1437591	85.85%	236892	14.15%	89196	37.65%
	p7	3700454	3243308	87.65%	457146	12.35%	158209	34.61%
	p8	6500730	5446068	83.78%	1054662	16.22%	397067	37.65%
	p9	2386840	1875333	78.57%	511507	21.43%	159734	31.23%
	p10	2849503	2313485	81.19%	536018	18.81%	137917	25.73%

C.

SCIFI_LIG384	GAGTTCAGACGTGTGCTCTTCCGATCT-NNNNNNNN-AAGTGATTAGCAA-TTTTTTTTTTTTTTTTTTTTTTTTTTTTTTVN
Well barcode	CAAGCAGAAGACGGCATACGAGAT[i7_barcode]GTGACTGGAGTTCAGACGTGTGCTCTTCCGATCT)

**D.**	Forward 5’ – 3’	Reverse 5’ – 3’

Exon 4-5<br>ENSG00000100836	ATGTTGGCAATGTGGACTATG	ACACGGTTGACTGAACCACA
Iso-201<br>ENST00000216727	GTTTTAACAGCAGGCCCCG	TCTTTTTTTCCTCTCTCTCCTCCTAATAC
Iso-202<br>ENST00000397276	CAACAGCCTTGTGGGAGGAT	CAAAAACCTGGGCACCACAC
Iso-207<br>ENST00000556821	CCCGACTGGCTTGATTCGG	CATGCTCGGCCATTTCCCT
HPRT1	TGGTCAGGCAGTATAATCCAAAGA	TCAAATCCAACAAAGTCTGGCTTA

A. Summary of data sets that were used in this study, transcripts' number that was used for APA-shift calculation and the number of significant APA-shift transcripts in each experiment.

B. RNAseq quality control in 7C and 1C protocols in OPMD mouse and cell line models, and in human muscles (controls and OPMD).

C. Primers used for library preparation.

D. Primers used for RT-qPCR

Table S3: Transcripts with APA-shift in mouse, cell line and human samples.

Table S4: List of muscle transcripts that were included in this study.

Table S5: expression levels of PABPN1 transcript isoforms in mouse and cell models.

Mouse Tibialis anterior

Transcript	Isoform	FVB_1	FVB_2	FVB_3	FVB_4	FVB_5	A17_1	A17_2	A17_3	A17_4	A17_5
ENSMUST00000022808	Iso-201	31.03	18.70	28.09	32.55	25.88	9.99	0.00	12.37	14.89	14.41
ENSMUST00000172557	Iso-208	34.75	23.04	29.19	34.23	23.16	13.58	0.00	10.49	18.69	11.31
ENSMUST00000116476	Iso-202	9.90	18.48	9.22	7.19	11.92	28.76	23.21	18.27	13.30	19.96
ENSMUST00000150975	Iso-207	4.95	11.95	8.06	4.49	10.21	20.37	19.34	14.21	10.45	13.65
ENSMUST00000172695	Iso-209	19.88	28.27	33.48	24.38	21.28	18.97	14.89	12.97	16.03	15.59

Muscle cell culture

Transcript	Isoform	WTA_1	WTA_2	WTA_3	D7E_1	D7E_2	D7E_3
ENSMUST00000022808	Iso-201	47.84	27.89	39.15	95.27	59.93	32.02
ENSMUST00000172557	Iso-208	60.77	32.16	30.19	113.91	65.50	42.11
ENSMUST00000116476	Iso-202	34.48	14.45	48.62	27.16	33.41	47.06
ENSMUST00000150975	Iso-207	25.08	14.45	24.31	18.10	27.84	43.70
ENSMUST00000172695	Iso-209	37.61	61.40	48.62	27.16	83.52	43.70

B. Supplementary figures

Figure S1: A flow-chat summary of the bioinformatic steps from RNA-seq to APA-shift calculation

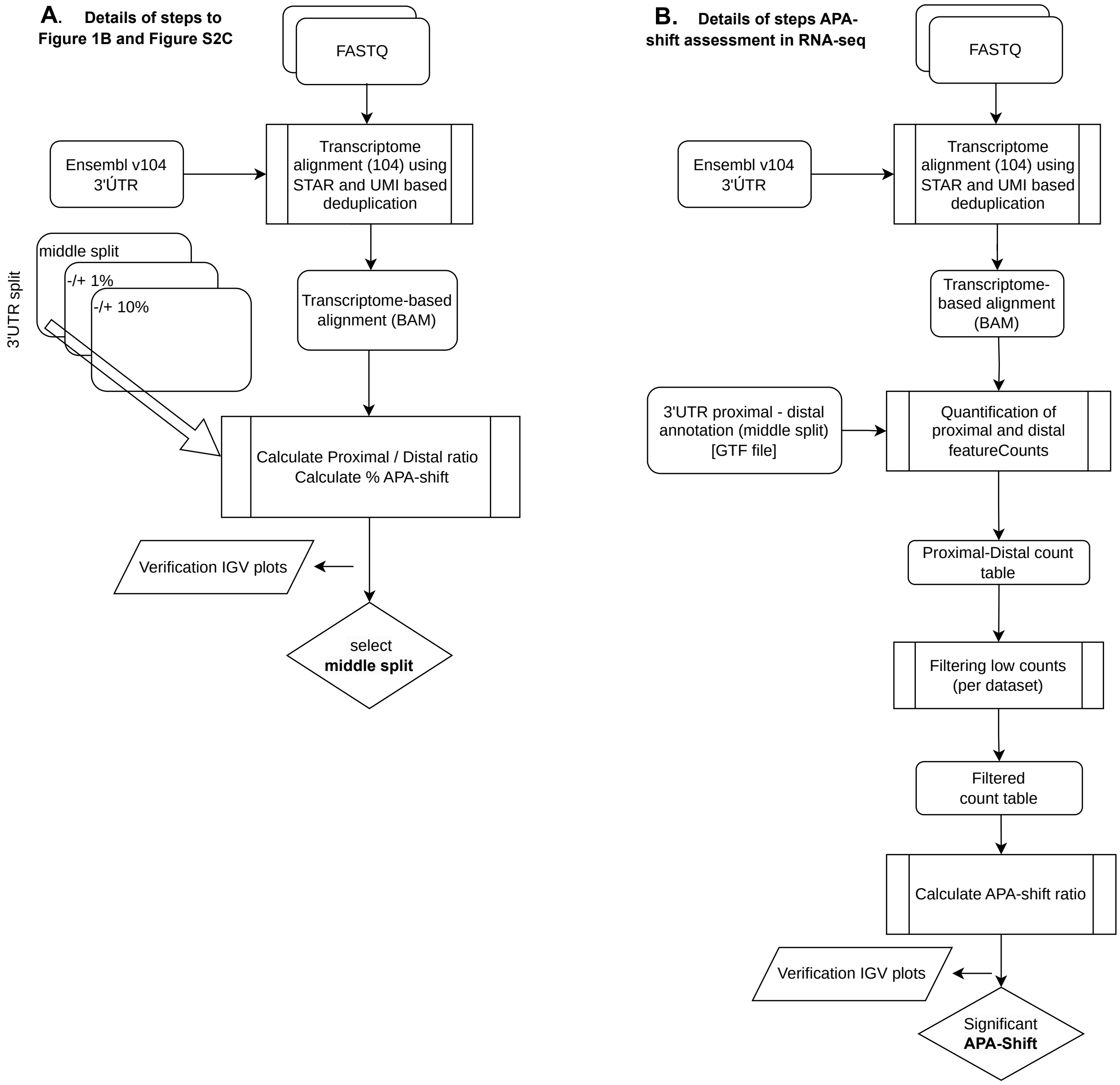

A. A flowchart of the steps to select the middle split in the 3'-UTR for APA-shift calculation. Results are in Fig. 1B and Figure S2C.

B. A flowchart of the steps to calculate APA shift from RNA-seq. The codes are in GIT.

Figure S2. 1C vs. 7C library prep results in mouse and cell OPMD models

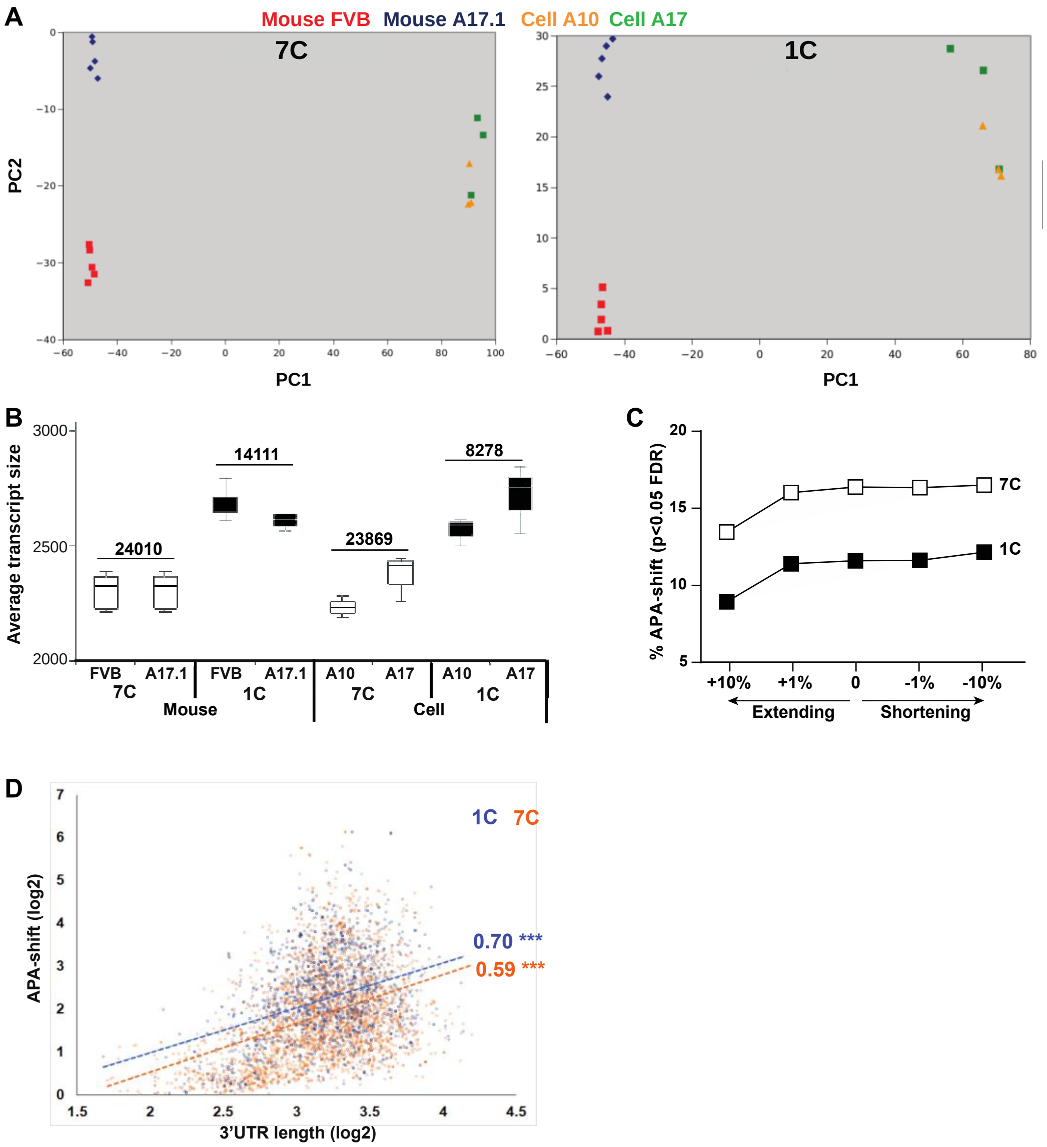

A. PCA plots of 7C or 1C RNAseq in mouse (FVB; A17.1) or mouse cells (A10; A17).

B. Boxplot of 7C and 1C average insert size in mouse and cell models. The number of transcripts passing exclusion criteria is depicted.

C. The percentage of transcripts with APA-shift (p<0.05, FDR) in mouse samples using different separation proximal and distal regions at the 3'UTR. 7C is denoted with open squares and 1C with close squares.

D. Dot plot of 3'UTR length vs APA-shift (log2) of transcripts with APA-shift<0.05, FDR, in 7C and 1C datasets. The linear regression line is depicted with a dashed line, and the slope and significance (*** p<0.0001) are shown next to the slope.

Figure S3. 1C vs. 7C APA-seq in an OPMD muscle cell model

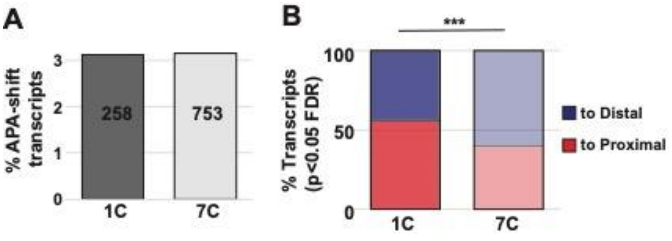

A. Bars show the percentage of transcripts with APA-shift from total transcripts in 1C or 7C datasets.

B. Bars show percentage of transcripts proximal (red) or distal (blow) APA-shift from the significant (p<0.05, FDR) APA-shift transcripts in 1C or 7C datasets. A statistical difference between 1C and 7C shift direction was assessed with the chi-square test; *** p<0.0001.

Figure S4: IGV and PAS-seq comparison in six transcripts

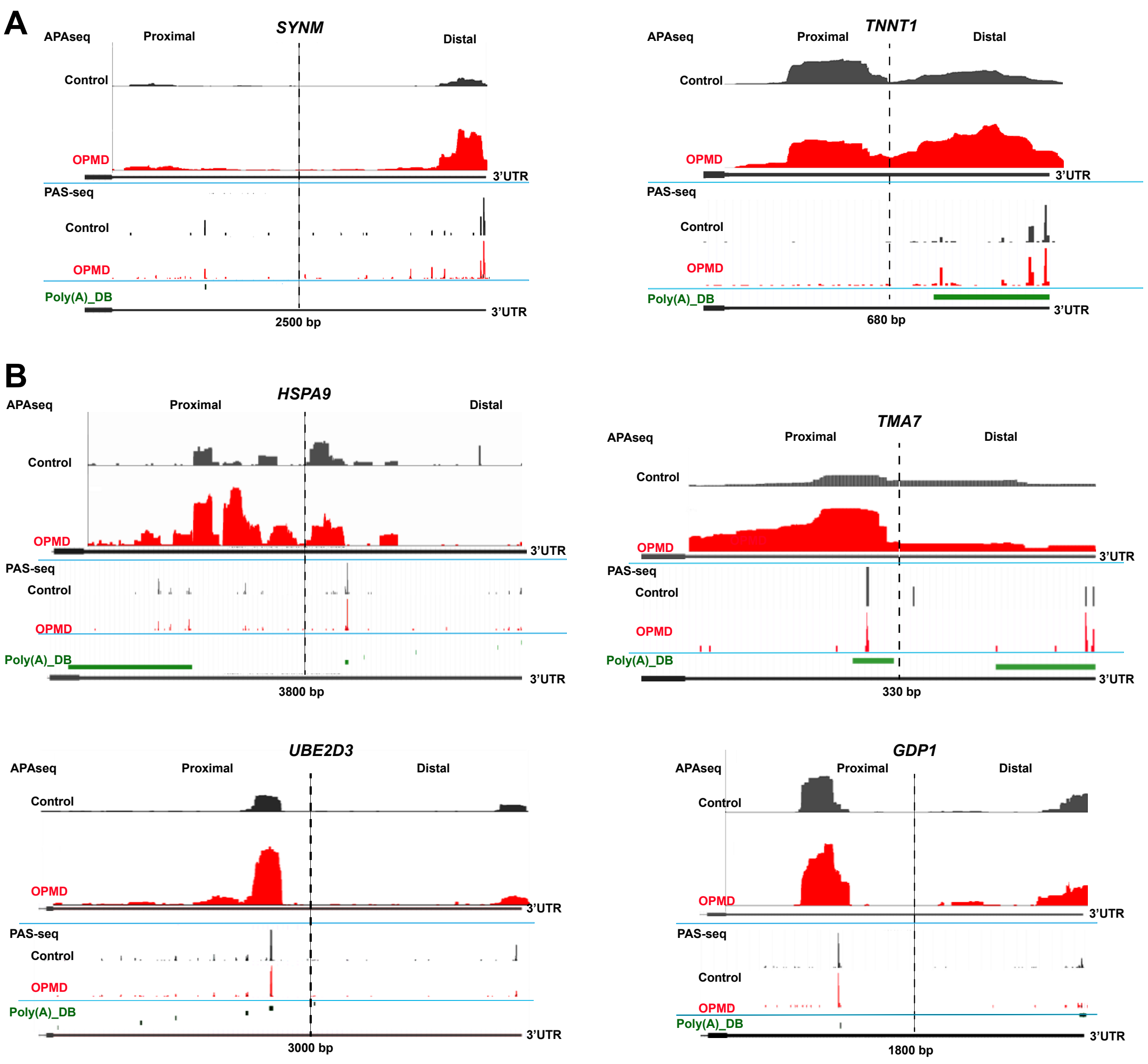

Transcripts examples are for shift to distal (A) or shift to proximal (B). Fer transcript, IGV in control (black) and OPMD (red) are on top followed by PAS-seq and PAS from poly(A)_DB (in green). The ensemble annotation 3'UTR is at the bottom, the length of the 3'UTR is depicted and a dashed line separates the proximal from distal regions.

Figure S5. PCA plots of APA-shift values in 1C samples

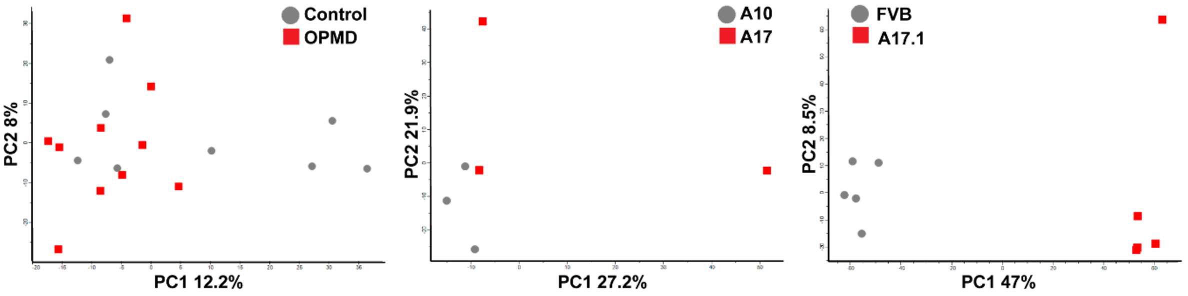

From left to right: human (controls and OPMD), muscle cell models (A10 and A17) , and mouse model (FVB and A17.1). The percentage of variance for the first two principal components (PC) is depicted.
